# Noncanonical regulation of imprinted gene *Igf2* by amyloid-beta 1–42 in Alzheimer’s disease

**DOI:** 10.1038/s41598-023-29248-x

**Published:** 2023-02-04

**Authors:** Emre Fertan, William H. Gendron, Aimée A. Wong, Gabrielle M. Hanson, Richard E. Brown, Ian C. G. Weaver

**Affiliations:** 1 Department of Psychology and Neuroscience, Dalhousie University, Halifax, NS B3H 4R2 Canada; 2 Department of Psychiatry, Dalhousie University, Halifax, NS B3H 4R2 Canada; 3 Department of Pathology, Dalhousie University, Halifax, NS B3H 4R2 Canada; 4 Brain Repair Centre, Dalhousie University, Halifax, NS B3H 4R2 Canada

**Keywords:** Neuroscience, Epigenetics in the nervous system, Molecular neuroscience, Neural ageing, Neurological disorders, Alzheimer's disease

## Abstract

Reduced insulin-like growth factor 2 (IGF2) levels in Alzheimer’s disease (AD) may be the mechanism relating age-related metabolic disorders to dementia. Since *Igf2* is an imprinted gene, we examined age and sex differences in the relationship between amyloid-beta 1–42 (*Aβ*_*42*_) accumulation and epigenetic regulation of the *Igf2/H19* gene cluster in cerebrum, liver, and plasma of young and old male and female 5xFAD mice, in frontal cortex of male and female AD and non-AD patients, and in HEK293 cell cultures. We show IGF2 levels, *Igf2* expression, histone acetylation, and *H19* ICR methylation are lower in females than males. However, elevated *Aβ*_*42*_ levels are associated with *Aβ*_*42*_ binding to *Igf2* DMR2, increased DNA and histone methylation, and a reduction in *Igf2* expression and IGF2 levels in 5xFAD mice and AD patients, independent of *H19* ICR methylation. Cell culture results confirmed the binding of *Aβ*_*42*_ to *Igf2* DMR2 increased DNA and histone methylation, and reduced *Igf2* expression. These results indicate an age- and sex-related causal relationship among *Aβ*_*42*_ levels, epigenomic state, and *Igf2* expression in AD and provide a potential mechanism for *Igf2* regulation in normal and pathological conditions, suggesting IGF2 levels may be a useful diagnostic biomarker for *Aβ*_*42*_ targeted AD therapies.

## Introduction

Alzheimer’s disease (AD) is the leading cause of dementia, in which synaptic loss and cerebral atrophy are associated with progressive cognitive decline and behavioural deficits^1^. In AD, dysregulation of amyloid-beta (*Aβ*) production and clearance leads to the appearance of amyloid plaques that primarily consist of *Aβ* peptides 1–40 (*Aβ*_*40*_) and 1–42 (*Aβ*_*42*_). Deposition of *Aβ*_*42*_ in the brain is associated with increases in oxidative stress, neuroinflammation, *tau* hyperphosphorylation, and synaptic dysfunction, as functional biomarkers that correlate with AD onset and progression^2–4^. *Aβ* aggregates, especially *Aβ*_*42*_, can translocate to neuronal nuclei and interact with regulatory elements of candidate genes involved in *Aβ* accumulation, such as amyloid precursor protein (*APP*), *β*-secretase, and apolipoprotein E (*ApoE*)^5–8^. We have reported that *Aβ*_*42*_ association with the thioredoxin interacting protein (*Txnip*) gene promoter region results in enhanced *Txnip* expression and TXNIP levels, which inhibits thioredoxin (TRX), and increases oxidative stress signalling that promotes DNA damage and cell death^9^, further demonstrating that intranuclear *Aβ*_*42*_ species can regulate gene expression.

Many metabolic disorders (e.g., diabetes, hypertension, obesity, and dyslipidemia) are risk factors for AD, linking abnormal liver function with cognitive deficits^10,11^. For example, in diabetic patients, impaired growth factor signaling, energy metabolism, inflammation, and insulin resistance promote hepatic cell death and liver damage^10^. Blood plasma can carry circulating DNA fragments (cell-free DNA, cfDNA) originating from cell death and degradation^12^. The cfDNA population in the bloodstream is heterogeneous and differs between individuals in normal ageing and pathological conditions such as AD^13^, suggesting that the biological properties of cfDNA fragments (including DNA and histone modification) may be a useful diagnostic biomarker for *Aβ*_*42*_ pathologies and AD progression.

Mouse models have been developed to study the mechanistic role of *Aβ* in AD neuropathology^14^ and behavioural deficits^15,16^. The five times familial mouse model of AD (5xFAD) shows one of the most aggressive *Aβ* pathologies due to the five mutations it carries on the *APP* [*V717I* (London), *I716V* (Florida) and *K670N/M671L* (Swedish)] and *presenilin 1* (*M146L* and *L286V*) genes^17^. The 5xFAD mice show cerebral *Aβ*_*42*_ accumulation as early as 2-months of age^17^, metabolic deficits and reduced body weight as early as 6-months of age^18^, followed by synaptic and neuronal loss as well as a decline in cognitive and motor performance by 9-months of age^19–22^.

Insulin-like growth factor 2 (IGF2) is one of the most abundant growth factors in the central nervous system (CNS)^23^ and is involved in the regulation of metabolism, tissue growth, and endocrine function^24,25^ in fetal growth and adulthood^26,27^. In patients with AD, reduced IGF2 levels have been reported in the hippocampus, cerebrospinal fluid, and blood plasma^28^. In mouse models of AD with *APP* mutations, increased IGF2 availability is associated with increased acetylcholine release and adult neurogenesis, and reduced amyloidosis and synaptic deficits^29,30^. Enhanced IGF2 availability also protects against hypoglycaemic damage in cultured hippocampal neurons^31^, enhances hippocampus-dependent memory consolidation^32,33^ and ameliorates age-related memory loss in rats^34^. Together, these findings suggest that IGF2 may be a useful biomarker for the metabolic deficits, pathological weight loss and cognitive decline of AD.

**Figure 1:**
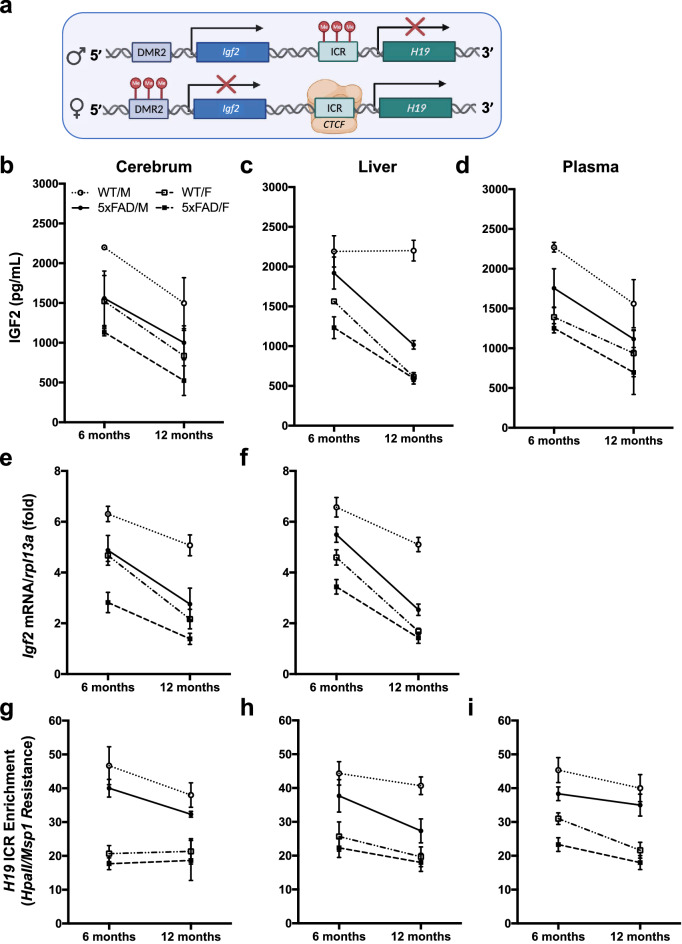
IGF2 levels, *Igf2* expression, and *H19* ICR methylation in the cerebrum, liver, and plasma from 6- and 12-month-old male and female WT and 5xFAD mice. (**a**) Schematic representation of the *Igf2/H19* gene cluster (see text for details). Parental-specific DNA methylation of the *H19* ICR defines the imprinted status of both *H19* and *Igf2. Igf2* expression on the *paternal allele* (upper row) is associated with *H19* ICR DNA methylation, *H19* silencing, and an unmethylated *Igf2* DMR2. Conversely, *Igf2* silencing on the *maternal allele* (lower row) is associated with an unmethylated *H19* ICR, transcription factor (CTCF) binding, *H19* expression, and *Igf2* DMR2 DNA methylation. (**b**–**d**) ELISA levels of IGF2, (**e**–**f**) RT-qPCR levels of *Igf2* mRNA, and (**g**–**i**) GlucMS-qPCR levels of *H19* ICR DNA methylation (5mC) in the cerebrum, liver, and plasma of female and male 5xFAD and WT mice at 6- and 12-months of age. Data are expressed as means + / − SEM. IGF2*/Igf2*, insulin-like growth factor 2; DMR2, differentially methylated region 2; ICR, imprinting control region; Me, DNA methylation; CTCF, CCCTC-binding factor; *rpl13a*, ribosomal protein *L13a*.; HpaII/Msp1, DNA restriction enzymes. See supplemental materials for results of statistical analyses.

IGF2 protein is encoded by *Igf2*, which was one of the earliest imprinted genes to be identified^35–37^. Genomic imprinting is the epigenetic silencing of an allele through DNA methylation, independent of the sex of the offspring^38,39^, resulting in the expression of a single allele from one parent, causing post-Mendelian parent-of-origin traits^40^. To date, 260 imprinted genes have been identified in mice and about 230 of these are conserved in humans^41^. The murine *H19*/*Igf2* gene locus is located on chromosome 7; *H19* is expressed from the maternal allele, and *Igf2* is expressed from the paternal allele^42,43^. *H19*/*Igf2* loci regulation (see Fig. 1a) is controlled by the DNA methylation status at well-characterized differentially methylated regions (DMRs), namely the *H19* imprinting control region (ICR), which is located upstream of the *H19* promoter (position: chr7: 142653816-142655810; see Fig. 1a). The unmethylated *H19* ICR on the maternally expressed mouse *Igf2* functions as a transcriptional insulator; CTCF binding to the *H19* ICR inhibits distal enhancers from binding to the *Igf2* promoter, resulting in *Igf2* silencing and expression of *H19* from the maternal allele. On the other hand, methylation of the *H19* ICR on the paternally expressed mouse *Igf2* gene inhibits CTCF binding, resulting in *H19* silencing and expression of *Igf2* from the paternal allele^43–47^.

Three DMRs have been identified in the mouse *Igf2* gene region. The maternally methylated DMR0 is in exon U1, whereas the paternally methylated DMR1 is positioned upstream of promoter 1, and DMR2 is in exon 6^48–51^ (Fig. 1a). In humans, the *H19*/*Igf2* locus is located on chromosome 11; and the regions homologous to mouse DMR0 and DMR2 are differentially methylated, whereas the region homologous to mouse DMR1 is unmethylated^52^. *Igf2* DMR1 and DMR2 knockout mouse studies revealed that DMR1 functions as a silencer^53^ and DMR2 as an activator^54^ of *Igf2* expression. Bi-allelic *Igf2* expression and hypermethylation at the *Igf2* DMR2 have been shown in patients with Beckwith–Wiedemann syndrome^52^, while loss of imprinting of *Igf2* DMRs has been shown in patients with Wilms tumour and colorectal cancer^55,56^. Consequently, loss of *Igf2* DMR imprinting (by DNA mutation or epimutation) and abnormalities in IGF2 function indicate the critical role of IGF2 in development and disease^57^. These findings suggest that *Aβ*_*42*_ and IGF2 potentially link peripheral biomarkers of liver functioning to central biomarkers related to AD progression, including *Aβ* aggregation, neural atrophy, and cognitive dysfunction.

Although genomic imprinting has been investigated in various brain regions of mouse models of AD^58,59^, it is unclear whether IGF2 expression is differently affected in symptomatic younger versus older transgenic AD mice. Moreover, it remains unclear how changes in *Igf2* expression are influenced by the accumulation and intranuclear role of *Aβ*_*42*_ as a transcription factor. We therefore compared *Aβ*_*42*_ and IGF2 levels in cerebrum, liver, and blood plasma from 6- and 12-month-old, male and female 5xFAD mice with age- and sex-matched B6SJLF1/J wild-type (WT) mice. The 5xFAD mice show initial weight loss and cognitive deficits at 6-months of age^18,60^, with severe deficits and increased mortality by 12-months of age, especially in males^61^, providing a useful model to study age-related expression patterns of *Igf2* and IGF2 in central, peripheral, and circulatory systems in response to *Aβ* accumulation. To determine whether the findings from the mouse model translated to human disease, we compared *Igf2* epigenetic regulation in frontal cortex from aged humans diagnosed with AD and non-AD controls. To examine whether *Aβ*_*42*_ association with the *Igf2* promoter region can directly mediate *Igf2* transcription, we treated the human-derived cell line, HEK293, with *Aβ*_*42*_ oligomers and harvested the cells at various time points to evaluate temporal changes in *Igf2* epigenetic status and expression.

## Materials and methods

### Mouse housing and breeding

All experimental procedures were performed in accordance with the guidelines of the Canadian Council on Animal Care and were approved by the Dalhousie University Committee on Laboratory Animals. Fifteen 5xFAD mice and 15 BSJLF1/J WT mice (6 males and 9 females of each genotype) were used in this study. All mice were bred in-house from pairs of male 5xFAD (B6SJLT-Tg (APPSwFlLon, PSEN1*M146L*L286V) 6799Vas/Mmjax; stock #034840) and female WT (C57BL/6JxSJL/J F1 mice; stock #100012) mice, originally purchased from the Jackson Laboratory (Bar Harbor, Maine). Pups were weaned at 21-days of age and housed in same sex, mixed genotype groups of 2–4 in transparent polyethylene cages (35 × 12 × 12 cm) with ad libitum food (Purina Rodent Chow, #5001) and tap water. Housing cages contained pine chip bedding and a polyvinyl chloride tube (5 cm diameter, 8 cm long) for enrichment and mice were provided with clean cages once a week. Mice were housed in a climate-controlled (20 ± 2 °C) vivarium under a 12:12 h reversed light/dark cycle with lights off between 09:30 and 21:30. Each mouse was individually marked with ear punches and genotyped for the *APP* and *PS1* transgenes using polymerase chain reaction (PCR) and the tissue samples from ear-punches. The research described here was conducted in compliance with the ARRIVE 2.0 Guidelines for Reporting Animal Research^62,63^.

### Tissue collection

At 5-weeks, 6-months, and 12-months of age mice were euthanized with sodium phenobarbital (200 mg/kg). After checking the toe-pinch reflex for signs of pain perception, blood was collected via cardiac puncture using 1 mL syringes washed with 10% ethylenediaminetetraacetic acid (EDTA). The blood was centrifuged at 1000*g* for 10 min at 4 °C and the plasma was collected. Following the cardiac puncture, mice were perfused with phosphate-buffered saline (PBS, 10%) solution for 2 min and livers and brains were harvested. Tissues and blood plasma were collected in 1.5-mL microcentrifuge tubes, frozen with dry ice, and stored at − 80 °C.

### Post-mortem human brain samples

All methods were carried out in accordance with the Tri-Council Policy Statement on Ethical Conduct for Research Involving Humans (Government of Canada). Informed, written consent forms were obtained for all subjects. Ethical approval was obtained from the Health Sciences Research Ethics Board (Halifax, Nova Scotia, Canada). Human brain samples and corresponding clinical and neuropathological diagnoses were provided by the Maritime Brain Tissue Bank (Halifax, Nova Scotia, Canada). A total of 12 frontal-cortex (orbitofrontal cortex, Brodmann’s area 11) samples from 6 AD and 6 non-AD patients (3 of each sex) were used in this study. The mean age of donors at the time of death was 76 years, which did not differ between the disease conditions or sexes (F_Disease_1, 8 = 2.32, p = 0.163; F_Sex_1, 8 = 0.41, p = 0.537). The clinical and neuropathological characteristics summarized in Table 1 show that cases 1–6 had no neuropathological hallmarks of AD sufficient for a clinical diagnosis, while cases 7–12 had progressive dementing illness and fulfilled the neuropathological criteria for AD^64^. During autopsy, the brains were removed and bisected through the midline. Half of the brain was fixed in formalin and used for neuropathologic diagnosis. The remaining (non-fixed) half was cut in 1–2 cm slabs, vacuum-sealed, frozen on dry ice and stored at − 80 °C until used in this study.

**Table 1:** Demographic data for human frontal cortex tissue samples.

Case	Sex	Age (y)	Post-mortem Interval (h)	Cause of death	Diagnosis
1	Male	73	68.5	Pulmonary embolism	Non-AD
2	Male	75	19.5	Renal failure	Non-AD
3	Male	75	5.0	Metastatic cholangiocarcinoma	Non-AD
4	Female	80	5.5	Complications from surgery	Non-AD
5	Female	71	7.0	Haemorrhage, cancer	Non-AD
6	Female	63	31.5	Cardio-renal failure	Non-AD
7	Male	72	6.5	Urosepsis	AD
8	Male	82	17.5	AD	AD
9	Male	71	22.0	Bowel obstruction	AD
10	Female	74	16.5	AD	AD
11	Female	85	48.0	AD, pernicious anaemia	AD
12	Female	91	37.0	Complications from hip fracture	AD

### Cell culture, Aβ_42_ peptide preparation, and treatment

Human embryonic kidney (HEK) 293 cells were grown as adherent cultures in Dulbecco’s Modified Eagle Medium (DMEM; Corning, VWR International Co., cat# 17-207-CV) supplemented with fetal bovine serum (FBS, 10%; Corning, VWR International Co., cat# 35-077-CV), 1 mM Sodium Pyruvate, 4 mM L-Glutamine, 25 mM d-Glucose, 100 units/mL penicillin, and 100 mg/mL streptomycin (VWR International Co., cat# K952-100ML) and maintained in a humidified incubator (5% CO_2_, 37 °C). Cultured cells were then counted using a haemocytometer and cell passaging was performed by washing residual media from cells using PBS (–Ca/–Mg; Gibco, Thermo Fisher Scientific Inc., cat# 14190144) and dissociating cells from the plate using Trypsin–EDTA (0.05%; Gibco, Thermo Fisher Scientific Inc., cat# 15400054). In each experiment, cultured cells were seeded into 6-well plates with 1.0 × 10^6^ cells per well. Human *Aβ*_*42*_ ‘click peptide’ (GenScript Biotech Inc., cat# RP10017-1) was dissolved in deionized H_2_O to make a 500 μM stock solution, which was further diluted to 5 μM with Dulbecco's PBS and incubated on a shaker (1.5 h, 300 rpm, 21 °C), as previously described^65^. Upon reaching 70% confluency, the HEK293 cultures received a single treatment of *Aβ*_*42*_ oligomer solution (500 nM) or saline vehicle control. The medium was replaced every 3 days and the cultures were harvested after 3-, 6-, and 9-days of incubation, the cell lysates were then collected for in vitro analysis. Treatments were repeated a minimum of three times using different cultures of HEK293 cells.

### Enzyme-linked immunosorbent assay (ELISA)

Protein isolation and ELISAs were performed as previously described^9,66^. ELISA was used to detect mouse IGF2 [detection range (DR), 15.6–1000 pg/mL; Cloud-Clone Corp; cat#: SEA051Mu], mouse and human reactive *Aβ*_*42*_ [DR, 7.4 to 250 pg/mL; BioLegend, San Diego, CA; cat#: 842401], human IGF2 [DR, 0.625–40 ng/mL; Cloud-Clone Corp; Cat#: SEA051HU, and human *Aβ*_*40*_ and *Aβ*_*42*_ [DR, 15.63 to 1000 pg/mL; Novus Biologicals Canada; cat# NBP2-69909 and NBP2-69913, respectively] according to the manufacturer’s protocols. The Novus Biologicals Canada's ELISA for beta-amyloid detection is specific for either human *Aβ*_*40*_ or human *Aβ*_*42*_ with minimal or no cross reactivity with other isoforms. Samples were loaded in triplicate alongside an eight-point standard curve in duplicate (with controls) in the 96-well microtiter plates supplied by each manufacturer. Detection was performed with horseradish peroxidase-labelled, Fc-specific IgG and read in the microplate reader at a wavelength of 620 nm (BioLegend mouse and human reactive *Aβ*_*42*_ kit) or 450 nm. The blank corrected levels of each target protein are reported in pg/mL.

### Real-time quantitative PCR (RT-qPCR)

The RNeasy Mini (QIAGEN Group, cat# 74004) and iScript cDNA Synthesis (Bio-Rad Laboratories Inc., cat# 1708891) kits were used to purify RNA and generate cDNA. Amplification reactions, containing SsoFast EvaGreen Supermix (Bio-Rad Laboratories Inc., cat# 1725201) with mouse or human *Igf2* and *rpl13a* primers (listed in Table 2), were run on the CFX Connect Real-Time PCR System (Bio-Rad Laboratories Inc.) as previously reported^9^. The 2^−∆∆CT^ method was used to analyze the relative changes in gene expression^67^.

**Table 2:** Primer sequences for Igf2 epigenetic regulation and expression assays.

Assay	Species	Gene	Forward primer (5′–3′)	Reverse primer (5′–3′)
RT-qPCR	Mouse	Igf2	GATACATGCTGCCCAAGTAACC	GACTGACAAAGATGGCCCATAG
		Rpl13a	ACAAGAAAAAGCGGATGGTG	TTCCGGTAATGGATCTTTGC
	Human	Igf2	TGGCATCGTTGAGGAGTGCTGTCTC	ACGGGGTATCTGGGGAAGTTGT
		Rpl13a	AAGGTGTTTGACGGCATCC	TACTTCCAGCCAACCTCGTGAG
GlucMS-qPCR	Mouse	H19 ICR	GGAACCGCCAACAAGAAAGT	GGTCTTTCCACTCACAACGG
	Human	H19 ICR	ACTCAAGTCCAGGCCAATTT	AAACGAATTGGCTGAGAAACAA
ChlP-qPCR	Mouse	H19 ICR	GATGCTAATGATCTCCGGC	CCATTCCGGAAGGGCTAA
		Igf2 DMR22	CCCAACCTCGGACCGT	TGAGCCCTCGAGGGGG
	Human	H19 ICR	ACTCAAGTCCAGGCCAATTT	AAACGAATTGGCTGAGAAACAA
		Igf2 DMR2	GCACGGAATTGGTTGTAGTTG	GTGACCCGGGACGTTTC

### Restriction enzyme-based DNA methylation analysis (GlucMS-qPCR)

The DNA methylation (5mC) and DNA hydroxymethylation (5hmC) levels in the mouse *H19* ICR were measured by restriction enzyme-based assay (EpiMark kit; New England Biolabs Inc., cat# E3317S) using the differential susceptibility of methylated and hydroxy-methylated DNA to cleavage by HpaII and MspI, as previously described^9^. Genomic DNA was extracted from cerebrum and liver tissues using the DNeasy Mini Kit (QIAGEN Group, cat# 69504), while circulating cfDNA was prepared from blood plasma using the QIAamp MinElute cfDNA Kit (QIAGEN Group, cat# 55284), according to the manufacturer’s protocols. The isolated DNA was treated with or without T4 Phage β-glucosyltransferase (T4-BGT; New England Biolabs Inc., cat#M0357S) to glucosylate 5hmC residues, rendering existing MspI sites non-cleavable. HpaII cleavage is prevented by 5mC and 5hmC. Glucosylated genomic DNA (100 ng) was digested with 10 U of either HpaII, MspI or no enzyme (8 h, 37 °C), followed by inactivation (20 min, at 80 °C). The HpaII- and MspI-resistant fraction was quantified by qPCR using mouse or human primers (listed in Table 2) designed around six HpaII/MspI sites (Fig. S1a and S2a), normalizing to the mock digestion control and two regions lacking HpaII/MspI sites. Resistance to MspI directly translates into percentage of 5hmC. 5mC levels were obtained by subtracting the 5hmC contribution from the total HpaII resistance.

### Chromatin immunoprecipitation assays (ChIP-qPCR)

ChIP-qPCR assays were performed as previously described^9,68^. Tissues were fixed with formaldehyde (1%) and the resulting crosslinked protein–DNA complexes were sonicated using the Q800R2 Sonicator system (Qsonica LLC) into 150–250 bp length fragments confirmed using the QIAxcel Advanced System (QIAGEN Group). Immunoprecipitation was then performed using rabbit polyclonal IgG antibodies against 5mC (AnaSpec Inc., cat# BI-MECY-0500), *Aβ*_*42*_ (MilliporeSigma Co., cat# AB5078P), CTCF (MilliporeSigma Co., cat# 07-729), H3K9Ac (Diagenode, cat# C15410004), H3K9me3 (Diagenode, cat# C15410193), as well as the control normal rabbit IgG (Santa Cruz Biotechnology Inc, cat# sc-2027). Post immunoprecipitation, the precipitated DNA–protein complexes were dissociated from the protein A conjugated Dynabeads (Invitrogen, Thermo Fisher Scientific Inc., cat# 10001D) and isolated using a Purelink PCR purification kit (Invitrogen, Thermo Fisher Inc. Scientific, cat# K310001) along with their respective total input controls (pre-immunoprecipitation DNA). RT-qPCR analyses were then performed using mouse and human primers (listed in Table 2) targeting the CTCF and *AβID* RE sites spanning the *H19* ICR (Fig. S1a and S2a) and *Igf2* DMR2 (Figs. 3a and 5a). Results are expressed as the fold change of the enrichment of the DNA detected under the treatment conditions against the DNA detected under the no treatment conditions. This was determined by dividing the signals obtained from the ChIP by the signals obtained from the total input control sample and normalizing for the DNA detected by the non-immune IgG (negative control). For sequential ChIP–reChIP experiments, the protein bound to the beads with the first antibody was incubated (30 min, 37 °C) twice with DTT (20 mM) and the combined elutes were suspended in ChIP dilution buffer, which was then immunoprecipitated (14 h, 4 °C) with the second antibody.

### Experimental design and statistical analyses

Each mouse was ear-punched and given a unique number. During the experiments, the investigators (E.F., G.M.H., I.C.G.W.) were blinded to sample genotype, sex, age, diagnosis, and treatment. The R Project Statistical Computing version 4.0.0 (2020-04-24)—"Arbor Day" was used for all statistical analyses, the graphs were generated in GraphPad Prism 7.0a for Mac OS X, and the cartoon figures were created with BioRender.com. Data from the mouse experiments were analysed within each tissue type (cerebrum, liver, plasma) between the genotypes (WT, 5xFAD), sexes (male, female) and ages (6-, 12-months). Human brain data were analysed between the disease status (non-AD, AD) and sexes (male, female). Data from the cell culture studies were analysed between the treatment conditions (vehicle treatment, *Aβ*_*42*_ treatment) and time points (3-, 6-, 9-days). All the analyses mentioned above were done using ANOVAs with Type 2 sums of squares and effect sizes were reported as partial eta squared (ηp^2^). *Post-hoc* group differences were determined by 95% confidence intervals (CI)^69,70^. Data for *Aβ*_*42*_ association with DNA were analyzed with Welch Two Sample t-tests and effect sizes were reported as Cohen’s d (*d*). Sample sizes were confirmed to be appropriate to achieve a type 2 error rate smaller than 0.05 for the majority of the measures by using the G*Power software^71^.

## Results

### Introductory overview

Because changes in IGF2 levels in the CNS and peripheral tissues have been associated with age-related cognitive decline and AD pathology in humans and mouse models^72^, we first measured IGF2 levels, *Igf2* expression, *H19* ICR methylation, CTCF binding, and histone modification in the cerebrum, liver, and blood plasma of 6- and 12-month-old, male and female 5xFAD and WT mice. The 5xFAD mice carry five mutations on two transgenes, which collectively increases *Aβ* production and the *Aβ*_*42*_ to *Aβ*_*40*_ ratio in the brain^17^. We therefore measured *Aβ*_*42*_ levels in the same three tissues to examine the age-related pattern of *Aβ*_*42*_ accumulation between 6- and 12-months of age in 5xFAD and WT mice. The next question concerned the temporal mechanism that potentially mediates changes in *Igf2* expression in the 5xFAD mice independent of *H19* ICR DNA methylation. In 5xFAD mice, the earliest timepoint *Aβ*_*42*_ accumulation has been reported in the brain is between 1.5- and 2-month of age^17,73^. We therefore used 5-week-old 5xFAD and WT mice to characterize *Igf2* epigenetic regulation and IGF2 levels prior to *Aβ*_*40*_ and *Aβ*_*42*_ accumulation. The results from the 5xFAD mice raised the question of whether changes in *Igf2* expression in AD patients are associated with changes in levels of *Aβ*_*42*_ binding to *AβID* regions on the *Igf2* promoter. We therefore characterized *Aβ*_*40*_ and *Aβ*_*42*_ accumulation, *Igf2/H19* epigenetic regulation, and IGF2 levels in the frontal cortex from aged individuals diagnosed with AD and non-AD patients. To further characterise the temporal epigenetic marks associated with *Aβ*_*42*_ binding to *AβID* regions on the *Igf2* promoter, we treated HEK293 cell cultures with a single dose of either *Aβ*_*42*_ oligomers or saline vehicle and then harvested the cultures at various time points for analysis of *Aβ*_*42*_ levels, *Igf2* epigenetic regulation, and IGF2 levels. The full results of statistical tests (including DoF, F- and p-values and CI) are given in the supplemental material.

### Age-related changes in IGF2 levels, *Igf2* expression and *H19* ICR methylation in 5xFAD and WT mice

Based on the results of ELISA assays, the levels of IGF2 in the cerebrum were significantly lower in 5xFAD than WT mice (Fig. 1b), significantly lower in females than males, and lower in 12-month than 6-month-old mice. While this age effect was significant for 5xFAD mice it was not significant for WT mice. In the liver, there was a genotype by sex by age interaction for IGF2 levels (Fig. 1c); all mice showed a decrease in IGF2 levels at 12-months of age except WT males which had significantly higher levels of IGF2 than all other 12-month-old mice. Circulating IGF2 levels in the blood plasma were significantly lower in 5xFAD than WT mice (Fig. 1d), significantly lower in 12-month than 6-month-old mice, and significantly lower in females than males. Together, these results show that genotype-, sex-, and age-related differences in IGF2 levels occur in the liver and blood plasma as well as in the cerebrum of 5xFAD and WT mice, indicating that IGF2 levels in peripheral tissues and the circulatory system are concomitant with those in the brain.

To determine whether these differences in IGF2 levels were associated with differences in *Igf2* expression, we performed RT-qPCR analyses in cerebrum and liver tissue. *Igf2* mRNA transcript levels in the cerebrum were significantly lower in 5xFAD than WT mice (Fig. 1e), significantly lower in 12-month than 6-month-old mice, and significantly lower in females than males. The *Igf2* mRNA levels in the liver were significantly lower in 5xFAD than WT mice and lower in females than males. There was also a significant genotype by sex by age interaction (Fig. 1f) as the *Igf2* mRNA levels were lower for all mice at 12-months than at 6-months of age, except for the WT males.

In the *H19*/*Igf2* locus, an ICR associated with a DMR2 sequence upstream of *H19* regulates the reciprocal expression of *Igf2* and *H19* (see Fig. 1a). Hypomethylation of the DMR2 on the maternal allele blocks enhancer driven *Igf2* transcription. To determine whether the differences in *Igf2* expression were associated with alterations in DNA methylation or DNA hydroxymethylation, we analysed 5mC and 5hmC levels in six distinct loci that are known to be differentially methylated in this region^48–51^. In both 6- and 12-month-old mice, the levels of *H19* ICR methylation (5mC) in the cerebrum were significantly lower in females than males (Fig. 1g), which reflects the reduced *Igf2* expression (Fig. 1e) and lower IGF2 levels (Fig. 1b). There was no significant effect of genotype or age on *H19* ICR 5mC levels in the cerebrum, suggesting that mechanisms independent of *H19* ICR methylation status regulate *Igf2* expression in aging 5xFAD mice. The *H19* ICR 5mC levels in the liver were significantly lower in 5xFAD than WT mice (Fig. 1h), which reflects the reduced *Igf2* expression levels (Fig. 1f) and lower IGF2 levels (Fig. 1c). Likewise, levels of *H19* ICR 5mC were significantly lower in the liver of females than males and significantly lower in the liver from 12-month than 6-month-old mice. The levels of DNA methylation (5mC) on the *H19* ICR of cfDNA in the blood plasma were significantly lower in 5xFAD than in WT mice, lower in females than males, and lower in 12-month than 6-month-old mice (Fig. 1i). The levels H19-ICR 5hmC levels did not differ between groups in the cerebrum, liver, and blood plasma (all p > 0.05; Table S1).

### *5xFAD*-associated tissue-specific variation in *H19* ICR methylation, CTCF binding and histone modification

Since hypermethylation of CpG dinucleotides of the *H19* ICR is associated with increased *Igf2* expression, while hypomethylation of CpG dinucleotides of the *H19* ICR is associated with CTCF binding and *Igf2* silencing, we tested the hypothesis that sex- and age-related differences in IGF2 levels, *Igf2* expression, and *H19* promoter hypomethylation in 5xFAD mice are associated with increased CTCF binding to the *H19* ICR (Fig. S1a). The results showed sex differences in DNA methylation (5mC) and in the levels of CTCF association with the *H19* ICR with females having lower 5mC and higher CTCF binding than males, and no genotype difference (Fig. S1b–d). Females also had higher levels of histone acetylation (H3K9Ac) following CTCF binding to the *H19* ICR than males (Fig. S1e–g), while males had higher levels of histone methylation (H3K9me3) following CTCF binding to the H19 ICR than females (Fig. S1h–j).

Overall, these findings show that IGF2 levels are associated with sex-differences in *H19* ICR DNA and histone modifications in the cerebrum, liver, and plasma, and that these enduring epigenetic marks potentially program chromatin accessibility and *Igf2* expression through CTCF association with the *H19* ICR in the cerebrum and liver. The lack of effect of genotype on group differences in CTCF association with the *H19* ICR methylation, CTCF binding, and histone modification in the cerebrum suggests that the changes in *Igf2* expression in 5xFAD mice may be caused by temporal mechanisms independent of *H19* ICR DNA methylation. Given the bidirectional communication between the nervous and liver systems (i.e., the liver-brain axis)^74^ and the potential role of *Aβ*_*42*_ as a transcription factor, this raised the question of whether changes in *Igf2* expression are linked to changes in *Aβ*_*42*_ deposition in the cerebrum and liver of 5xFAD mice.

### 5xFAD-associated tissue-specific variation in Aβ_42_ levels

**Figure 2:**
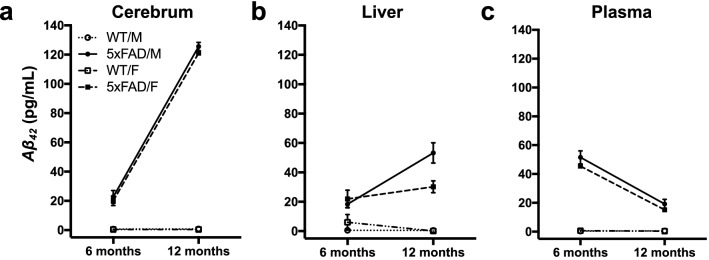
ELISA levels of *Aβ*_*42*_ in the cerebrum, liver, and plasma of male and female, 5xFAD and WT mice at 6- and 12-months of age. Data are expressed as means + / − SEM. *Aβ*_*42*_, amyloid beta 1–42.

The results of ELISA analyses show that the levels of *Aβ*_*42*_ were higher in the cerebrum of 5xFAD than WT mice and increased with age in 5xFAD but not in WT mice, resulting in an age by genotype interaction, with no sex difference (Fig. 2a). Levels of *Aβ*_*42*_ were higher in the liver of 5xFAD than WT mice and increased significantly with age in 5xFAD mice but not in WT mice resulting in an age by genotype interaction. There was also a sex by age interaction, as levels of *Aβ*_*42*_ in the liver increased more in males from 6- and 12-months of age than females (Fig. 2b). The levels of *Aβ*_*42*_ were higher in the blood plasma of 5xFAD than WT mice, and decreased with age in 5xFAD mice, but not in WT mice. There was no sex difference in levels of *Aβ*_*42*_ in the blood plasma (Fig. 2c). These results confirm the earlier findings^17^ of elevated *Aβ*_*42*_ levels in the 5xFAD mice in an age-dependent manner and show that this increase occurs in the liver as well as the brain.

### ***5xFAD***-associated tissue-specific variation in ***Igf2*** DMR2 methylation, ***Aβ***_***42***_ binding and histone modification

**Figure 3:**
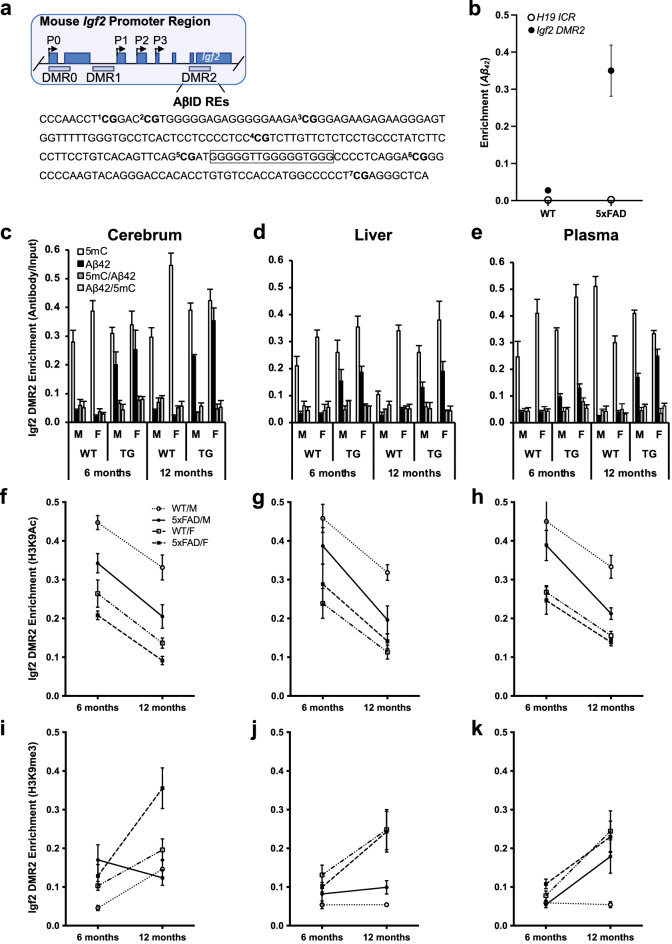
Epigenetic marks associated with *Aβ*_*42*_ binding to *Igf2* DMR2 in mice. (**a**) Schematic representation of the mouse *Igf2* promoter region (also see Fig. 1a). Mouse *Igf2* has four promoters (P, 0–3) and three DMRs (0–2). Beneath is shown the *Igf2* DMR2 DNA sequence we analyzed, with the location of seven CpG sites (bold) relative to the predicted *Aβ*_*42*_ interacting domain (A*β*ID; boxed area). (**b**) ChIP-qPCR analyses of *Aβ*_*42*_ association with *H19* ICR and *Igf2* DMR2 in cerebrum from 12-month-old 5xFAD mice. ChIP- and double ChIP-qPCR analyses of DNA methylation (5mC) and *Aβ*_*42*_ association with *Igf2* DMR2 in (**c**) cerebrum, (**d**) liver, and (**e**) plasma of male and female, 5xFAD and WT, 6- and 12-month-old mice. ChIP-qPCR analyses of H3K9Ac (**f**–**h**) and H3K9me3 (**i**–**k**) association with *Igf2* DMR2 in cerebrum, liver, and plasma of male and female, 5xFAD and WT, 6- and 12-month-old mice. Data are expressed as means − / + SEM. *Igf2*, insulin-like growth factor 2; DMR2, differentially methylated region 2; ICR, imprinting control region; 5mC, 5-methylcytosine; *Aβ*_*42*_, amyloid beta 1–42; H3K9Ac, histone 3 lysine-9 acetylation; H3K9me3, histone 3 lysine-9 tri-methylation.

Results from neural (SH-SY5Y) cell cultures^75^ and the triple transgenic mouse model of AD^9^ provide evidence that *Aβ* peptides, especially *Aβ*_*42*_, can translocate to neuronal nuclei and bind to the *Aβ* interacting domain (*AβID*) within gene regulatory and promoter elements to alter gene expression. This raises the question of whether changes in *Igf2* expression in 5xFAD mice are associated with changes in levels of *Aβ*_*42*_ binding to *AβID* regions on the *Igf2* promoter. Three DMRs have been identified in the mouse *Igf2* promoter region (Fig. 3a). The paternally methylated DMR2 functions as a transcriptional activator of *Igf2* expression when hypomethylated and contains *AβID* response elements (REs)^54^. We first performed ChIP-qPCR analyses with an antibody toward *Aβ*_*42*_ to test the specificity of the association of *Aβ*_*42*_ with the potential *AβID* region of *Igf2* DMR2 in cerebrum from 12-month-old mice and found that *Aβ*_*42*_ association with *Igf2* DMR2 was significantly higher than with the *H19* ICR that did not include the potential *AβID* region in 5xFAD mice (Fig. 3b). This raises the question of whether the decrease in IGF2 levels and *Igf2* expression in the 5xFAD mice is associated with increased *Aβ*_*42*_ binding with *Igf2* DMR2. To test this, we performed ChIP analysis with antibodies toward 5mC and *Aβ*_*42*_ with the *AβID* region of *Igf2* DMR2 in cerebrum, liver, and blood plasma from 6- and 12-month-old male and female 5xFAD and WT mice.

Levels of DNA methylation on the *AβID* region of *Igf2* DMR2 in the cerebrum showed no genotype difference but were significantly higher in 12-month than 6-month-old mice, and there was a sex by genotype interaction. While female WT mice had higher levels of *Igf2* DMR2 methylation in the cerebrum than male WT mice, there was no sex difference in 5xFAD mice (Fig. 3c). However, levels of *Aβ*_*42*_ association with the *AβID* region of *Igf2* DMR2 in the cerebrum were significantly higher in 5xFAD than WT mice, with no effect of sex or age (all p > 0.05; Fig. 3c). Levels of DNA methylation on the *AβID* region of *Igf2* DMR2 in the liver were significantly higher in 5xFAD than WT mice, and were significantly higher in females than males, with no effect of age (Fig. 3d). Levels of *Aβ*_*42*_ association with the *AβID* region of *Igf2* DMR2 in the liver were significantly higher in 5xFAD than WT mice, with no effect of sex or age (all p > 0.05; Fig. 3d). There was no effect of genotype on levels of *Igf2* DMR2 methylation of cfDNA fragments from the plasma, but there was a sex by age interaction: females had lower levels at 12-months than 6-months of age, while males had higher levels at 12-months than 6-months of age (Fig. 3e). The 5xFAD mice showed significantly higher *Aβ*_*42*_ binding in the plasma than WT mice, and there were genotype by age and genotype by sex interactions: *Aβ*_*42*_ association with the *Igf2* promoter increased in 5xFAD mice as they aged, and the increase was greater in 5xFAD females than males (Fig. 3e), suggesting that cfDNA epigenetic marks at transcription factor binding sites in plasma also reflect age-related AD pathologies. The double ChIP analyses showed low levels *Igf2* DMR2 enrichment in lanes labelled 5mC/*Aβ*_*42*_ and *Aβ*_*42*_/5mC, with no significant genotype, sex, or age differences in cerebrum, liver, or blood plasma (all p values > 0.05; Fig. 3c–e).

*Aβ*_*42*_ binding at the *Igf2* DMR2 is associated with *Igf2* silencing, suggesting that an association of *Aβ*_*42*_ with specific changes in chromatin through histone modification could also influence *Igf2* DMR2 activity. To test this hypothesis, we performed ChIP-qPCR analyses measuring histone acetylation (H3K9Ac) and histone methylation (H3K9me3) within the *AβID* region in 5xFAD and WT mice. Levels of H3K9Ac association with the *AβID* region of *Igf2* DMR2 in the cerebrum were significantly lower in 5xFAD than WT mice, lower in females than males, and lower in 12-month than 6-month-old mice (Fig. 3f). While there was no genotype effect, there was a significant genotype by sex interaction for H3K9Ac association with the *AβID* region of *Igf2* DMR2 in the liver: while males had overall higher levels than females, the sex difference was only significant within the WT mice and not the 5xFAD mice. Moreover, the levels of H3K9Ac association were lower in 12-month than 6-month-old mice (Fig. 3g). Levels of H3K9Ac association with the *AβID* region of *Igf2* DMR2 in the blood plasma were also significantly lower in 5xFAD than WT mice, lower in females than males, and lower in 12-month than 6-month-old mice (Fig. 3h).

The overall levels of H3K9me3 association with the *AβID* region of *Igf2* DMR2 in the cerebrum were higher in 5xFAD than WT mice, higher in 12-month than 6-month-old mice, and higher in females than males, but there was a genotype by sex by age interaction as 5xFAD males had a decrease with age whereas all other groups showed an increase with age (Fig. 3i). In the liver, there was no significant genotype difference for H3K9me3 association, but there was a sex by age interaction; females showed an increase from 6- to 12-months of age but males did not (Fig. 3j). Levels of H3K9me3 association with the *AβID* region of *Igf2* DMR2 in the blood plasma were significantly higher in females than males, and higher in 12-month than 6-month-old mice, with no effect of genotype (Fig. 3k). These results suggest that *Aβ*_*42*_ association with the *Igf2* DMR can alter the chromatin structure and decrease *Igf2* expression by changing both histone acetylation and methylation in a sex, age, and tissue specific manner.

### IGF2 levels, *Igf2* expression, and epigenetic status of the *H19/Igf2* locus in 5-week-old 5xFAD and WT mice

There was no difference in *Aβ*_*40*_ or *Aβ*_*42*_ accumulation in 5-week-old 5xFAD compared to WT mice, and no significant genotype differences in IGF2 levels, *Igf2* mRNA levels, or epigenetic status of the *H19/Igf2* locus (Table S2). These findings suggest that *Aβ*_*40*_ and *Aβ*_*42*_ accumulation either precede, or occur concomitantly with, the tissue-specific alterations in *Igf2* epigenetic regulation and IGF2 levels observed in 6- and 12-month-old 5xFAD mice.

### Frontal cortex IGF2 and *Igf2* mRNA reduction and *H19* ICR methylation in AD patients

**Figure 4:**
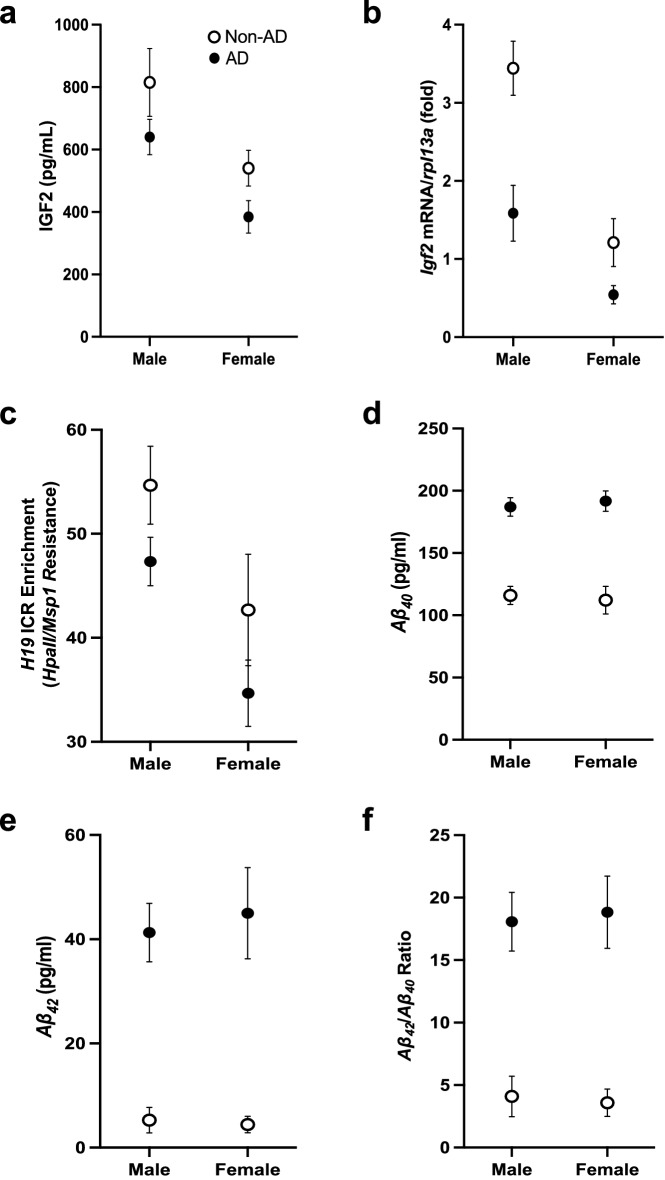
IGF2 levels, *Igf2* expression, *H19* ICR methylation, and *Aβ*_*40*_ and *Aβ*_*42*_ levels in the frontal cortex of male and female AD and non-AD patients. (**a**) ELISA levels of IGF2, (**b**) RT-qPCR levels of *Igf2* mRNA, (**c**) GlucMS-qPCR levels of *H19* ICR DNA methylation (5mC), and ELISA levels of (**d**) *Aβ*_*40*_, (**e**) *Aβ*_*42*_, and (**f**) the *Aβ*_*42*_/*Aβ*_*40*_ ratio in the frontal cortex of male and female, AD and non-AD patients. Data are expressed as means + / − SEM. IGF2*/Igf2*, insulin-like growth factor 2; *rpl13a*, ribosomal protein *L13a; HpaII*/Msp1, DNA restriction enzymes*; Aβ*_*40*_, amyloid beta 1–40;* Aβ*_*42*_, amyloid beta 1–42.

To determine whether the findings from the 5xFAD mouse model translated to humans, we compared *Igf2* epigenetic regulation and IGF2 levels in the frontal cortex from aged humans diagnosed with AD and age-matched non-AD controls. As in 5xFAD mice, IGF2 levels in the frontal cortex were lower in AD than non-AD patients and lower in females than males (Fig. 4a; Table S3). In agreement with this, *Igf2* mRNA levels in the frontal cortex were lower in AD than non-AD patients, and lower in females than males (Fig. 4b; Table S3).

To determine whether the differences in *Igf2* expression were associated with alterations in DNA methylation and hydroxymethylation we analysed 5mC and 5hmC levels in six distinct loci that are known to be differentially methylated in the *H19* ICR (Fig. S2a). The levels of *H19* ICR 5mC in the frontal cortex were significantly lower in females than males (Fig. 4c) but did not differ between AD and non-AD patients. Levels of *H19* ICR 5hmC did not differ between the disease conditions or sexes (all p > 0.05; Table S3). Results of ELISA analyses showed that AD patients had higher levels of *Aβ*_*40*_ (Fig. 4d), *Aβ*_*42*_ (Fig. 4e), and a higher *Aβ*_*42*_/*Aβ*_*40*_ ratio (Fig. 4f) than non-AD patients. There were no sex differences in any of these measures (all p > 0.05; Table S3). Together these results demonstrate that *Aβ*_*42*_ accumulation in the frontal cortex is associated with *Igf2* expression in AD pathology in human patients.

### *H19* ICR DNA methylation, CTCF binding, and histone modifications in AD patients

We tested the hypothesis that the variations in frontal cortex levels of IGF2 and *Igf2* mRNA transcripts, and *H19* promoter hypomethylation in AD are associated with altered CTCF association with the *H19* ICR (Fig. S2a). Overall, these findings (see Fig. S2 and Table S3 for statistical analyses) show that males had higher levels of *H19* ICR DNA methylation than females, with no effect of AD, and that CTCF association was higher in non-AD females than non-AD males (Fig. S2b), with no AD- or sex-differences in H3K9ac or H3K9me3 (Fig. S2c,d).

### AD status- and sex-specific variation in ***Igf2*** DMR2 methylation, ***Aβ***_***42***_ binding, and histone modification in humans

**Figure 5:**
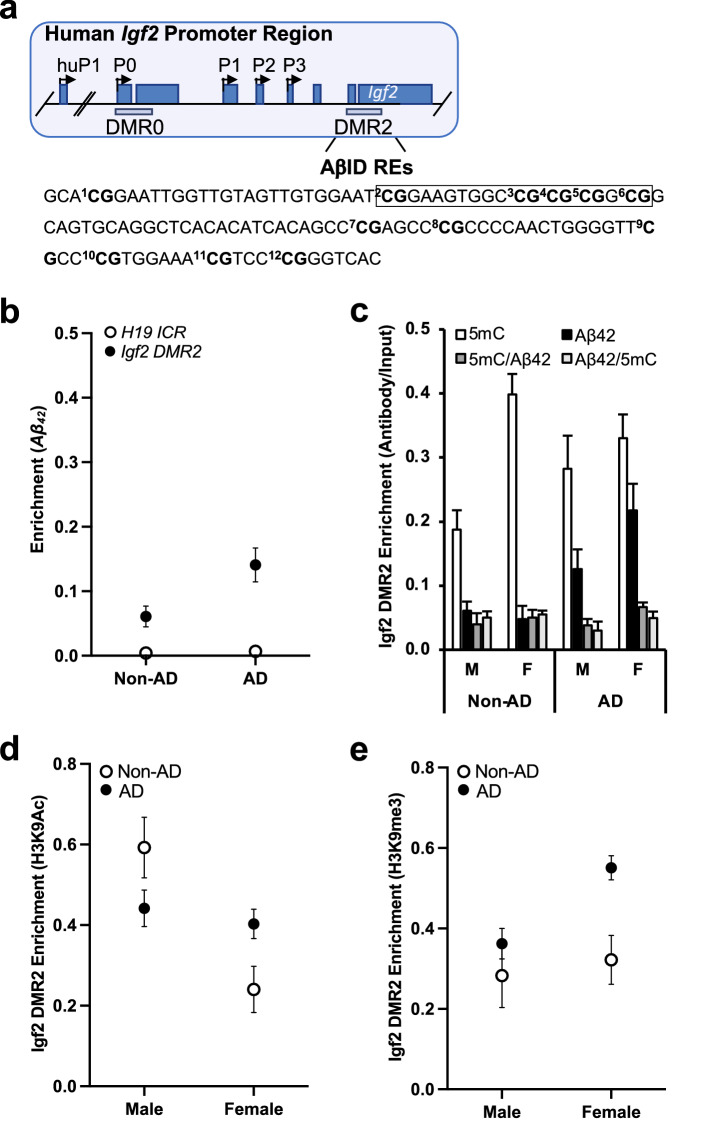
Epigenetic marks associated with *Aβ*_*42*_ binding to *Igf2* DMR2 in human frontal cortex. (**a**) Schematic representation of the human *Igf2* promoter region (also see Fig. 1a). Human *Igf2* has five promoters (human P1, HuP1; and P, 0–3) and two DMRs (0 and 2). There is no mouse homologue for HuP1; however, P0–3, DMR0 and DMR2 are homologous in human and mouse. Beneath is shown the *Igf2* DMR2 DNA sequence we analyzed, with the location of twelve CpG sites (bold) relative to the predicted *Aβ*_*42*_ interacting domain (A*β*ID; boxed area). (**b**) ChIP-qPCR analyses of *Aβ*_*42*_ association with *H19* ICR and *Igf2* DMR2 in AD and non-AD patients. ChIP- and double ChIP-qPCR analyses of DNA methylation (5mC) and *Aβ*_*42*_ association with the *Igf2* DMR2 in male and female, AD and non-AD patients. ChIP-qPCR analyses of H3K9Ac (**d**) and H3K9me3 (**e**) association with *Igf2* DMR2 in male and female, AD and non-AD patients. Data are expressed as means + / − SEM.

Two DMRs have been identified in the human *Igf2* promoter region (Fig. 5a). Using ChIP-qPCR analyses with an antibody toward *Aβ*_*42*_ we found that there was significantly greater *Aβ*_*42*_ association with the *Igf2* DMR2 sequence containing the potential *AβID* region in frontal cortex of AD patients than there was with the *H19* ICR that did not include this *AβID* region (Fig. 5b; Table S3). Using ChIP-qPCR analyses with antibodies toward 5mC and *Aβ*_*42*_ we found that levels of *Igf2* DMR2 methylation (5mC) in the frontal cortex were not significantly different between AD and non-AD patients, but were significantly higher in females than males (Fig. 5c). Conversely, *Aβ*_*42*_ association with *Igf2* DMR2 was significantly higher in the frontal cortex from AD compared to non-AD patients, but there was no sex difference (Fig. 5c). Similar to the results in mice, the double ChIP assays demonstrated that *Aβ*_*42*_ did not strongly bind to methylated DNA (evidenced by low levels *Igf2* DMR2 enrichment in lanes labelled 5mC/*Aβ*_*42*_ and *Aβ*_*42*_/5mC; Fig. 5c). The ChIP-qPCR analyses measuring H3K9Ac and H3K9me3 within the *AβID* region found that the levels H3K9Ac association with *Igf2* DMR2 in the frontal cortex were higher in female than male non-AD patients but not in AD patients (Fig. 5d). Conversely, H3K9me3 association with *Igf2* DMR2 in the frontal cortex was significantly higher in AD than non-AD patients, with no significant sex difference (Fig. 5e).

### Effects ***Aβ***_***42***_ exposure on ***Igf2*** promoter regulation in HEK293 cell culture

**Figure 6:**
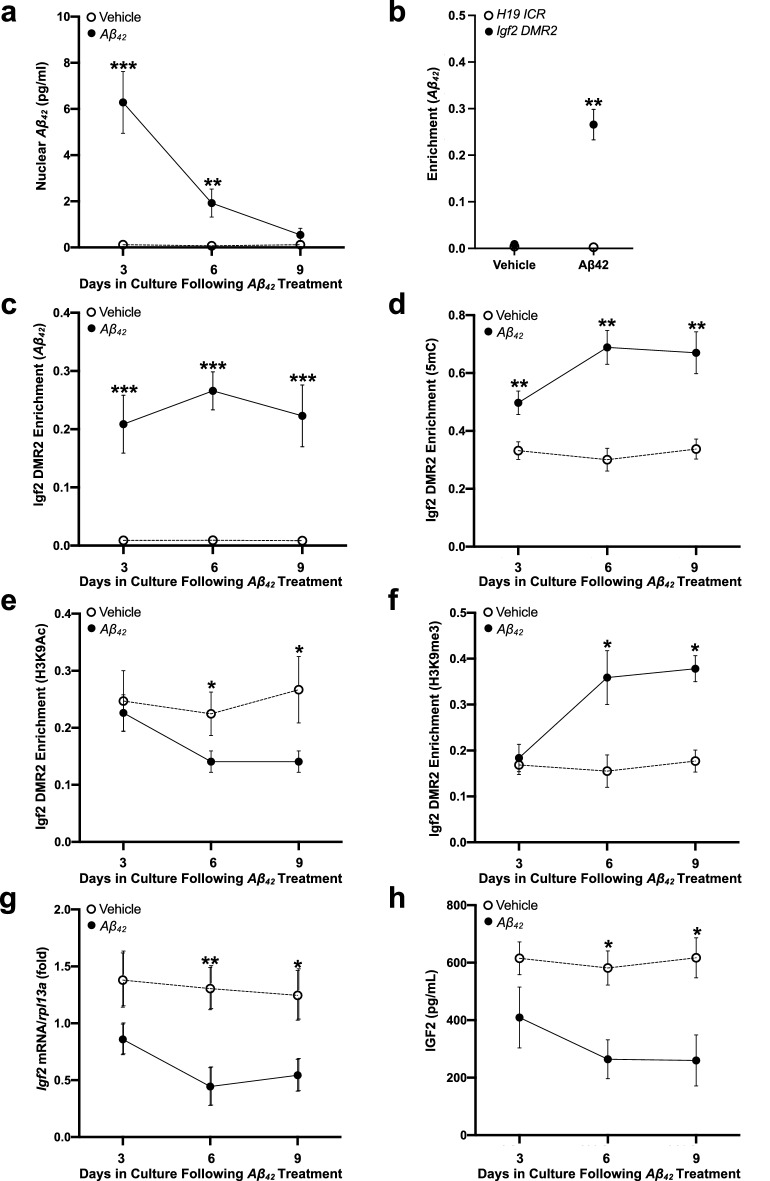
Effects of *Aβ*_*42*_ on *Igf2* regulation and IGF2 levels in HEK239 cells. Cell cultures received a single treatment of *Aβ*_*42*_ oligomer solution or saline vehicle, which were replaced every 3 days (removing *Aβ*_*42*_ from the media), and the cells were harvested after 3-, 6-, or 9-days for in vitro assays. (**a**) ELISA levels of *Aβ*_*42*_ from nuclear extracts in *Aβ*_*42*_-treated cultures. (**b**) ChIP-qPCR analyses of *Aβ*_*42*_ association with *H19* ICR and *Igf2* DMR2 (containing the *AβID*) 6-days following *Aβ*_*42*_-treatment. ChIP-qPCR analyses of (**c**) *Aβ*_*42*_, (**d**) DNA methylation (5mC), (**e**) H3K9Ac, and (**f**) H3K9me3 association with *Igf2* DMR2 in *Aβ*_*42*_-treated cultures. (**g**) RT-qPCR analyses of *Igf2* mRNA levels and (**h**) ELISA analysis of IGF2 levels in *Aβ*_*42*_-treated cultures. Data are expressed as means + / − SEM (*p < 0.05; **p < 0.01; ***p < 0.001).

Results of the ELISA analyses found that levels of *Aβ*_*42*_ in the cell nuclear fractions were higher in the *Aβ*_*42*_-treated than the vehicle-treated HEK293 cells as a function of the number of days the cells spent in culture following *Aβ*_*42*_ treatment (Fig. 6a; Table S4). Three-days following treatment, the magnitude of the difference in *Aβ*_*42*_ levels between *Aβ*_*42*_- and vehicle-treated cultures was greatest. While still significant, 6-days after treatment the magnitude of the difference in *Aβ*_*42*_ levels between *Aβ*_*42*_- and vehicle-treated cultures was reduced and was not significant 9-days following *Aβ*_*42*_ treatment (Fig. 6a). These results suggest that exogenous *Aβ*_*42*_ can enter the cell nucleus, followed by temporal removal (clearing). The ChIP-qPCR analyses with an antibody toward *Aβ*_*42*_ showed that in the *Aβ*_*42*_ treated cultures, there was significantly greater *Aβ*_*42*_ binding with *Igf2* DMR2 than there was with *H19* ICR, while there was no difference in the vehicle-treated cultures (Fig. 6b). The levels of *Aβ*_*42*_ binding to *Igf2* DMR2 were significantly higher in *Aβ*_*42*_-treated cultures than control cultures at all time points (Fig. 6c), as were the levels of *Igf2* DMR2 DNA methylation (Fig. 6d).

Levels of *Igf2* DMR2 histone acetylation were decreased in the *Aβ*_*42*_-treated cultures compared to control cultures after 6 and 9 days (Fig. 6e), while levels of *Igf2* DMR2 histone methylation increased as a function of the number of days the cells spent in culture following *Aβ*_*42*_ treatment (Fig. 6f). The levels of *Igf2* DMR2 histone methylation in *Aβ*_*42*_-treated and vehicle-treated cultures were not significantly different after 3-days of *Aβ*_*42*_ treatment, while after 6- and 9-days they were significantly higher in *Aβ*_*42*_-treated than control cultures. Levels of *Igf2* mRNA were significantly lower in *Aβ*_*42*_-treated cultures at 6- and 9-days, but not at 3-days following *Aβ*_*42*_ treatment (Fig. 6g). Likewise, levels of IGF2 were significantly lower in *Aβ*_*42*_-treated than control cultures at 6- and 9-days, but not at 3-days after *Aβ*_*42*_ treatment (Fig. 6h). Together, these results suggest a causal relationship between *Aβ*_*42*_ accumulation, *Aβ*_*42*_ binding and DNA methylation on the *Igf2* DMR2, combined with histone modifications (deacetylation and methylation), leading to a stable reduction of *Igf2* expression and IGF2 levels (Table S4). Stable association of *Aβ*_*42*_ with the *Igf2* DMR2 provides a potential mechanism underlying the temporal stability of altered *Igf2* expression patterns in 5xFAD mice and humans diagnosed with AD.

## Discussion

**Figure 7:**
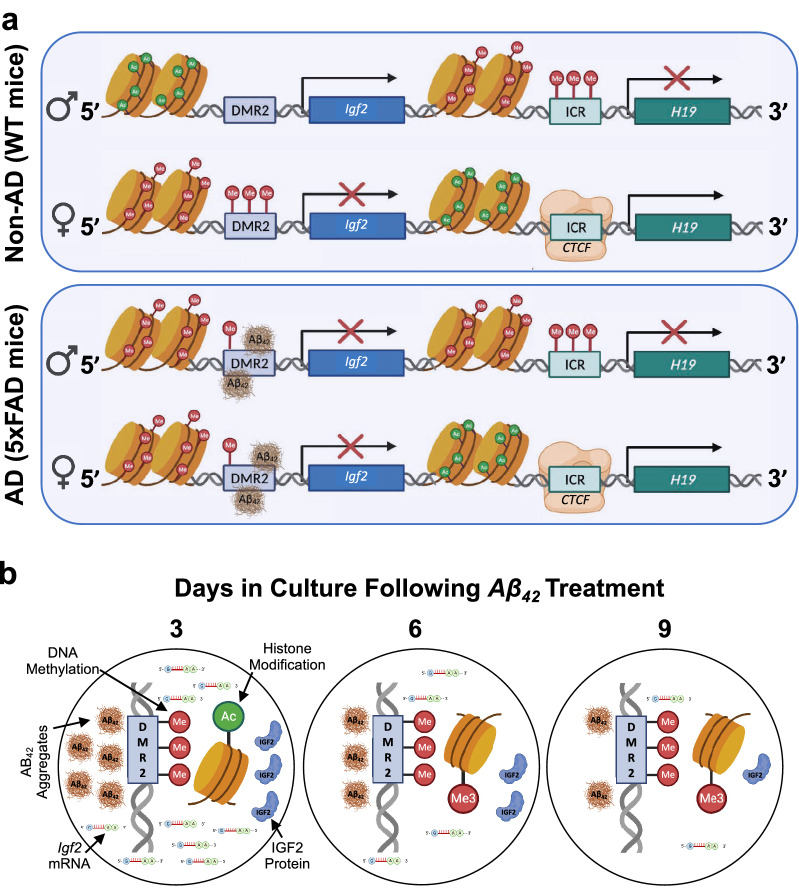
Dynamics of histone modification, CpG methylation, and transcription factor binding that control *Igf2* expression associated with *Aβ*_*42*_ accumulation in AD. (**a**) *In Non-AD (WT mice)*: enhanced *Igf2* expression on the *paternal allele* (upper row) is associated with histone hypermethylation (H3K9me3), DNA hypermethylation (5mC), and reduced transcription factor (CTCF) binding to the *H19* ICR, with concomitant histone hyperacetylation (H3K9Ac) and DNA hypomethylation of *Igf2* DMR2. Conversely, attenuated *Igf2* expression on the *maternal allele* (lower row) is associated with histone hyperacetylation (H3K9Ac), DNA hypomethylation, and increased transcription factor (CTCF) binding to the *H19* ICR, with concomitant histone hypermethylation (H3K9me3), and DNA hypermethylation (5mC) of *Igf2* DMR2. In AD (*5xFAD mice*): as in WT mice, attenuated *Igf2* expression on the *paternal allele* (upper row) is associated with histone hypermethylation (H3K9me3), and DNA hypermethylation (5mC); *however*, enhanced transcription factor (*Aβ*_*42*_) binding to *Igf2* DMR2 is associated with histone hypoacetylation (H3K9Ac), histone hypermethylation (H3K9me3), DNA hypermethylation (5mC) and attenuation of *Igf2* expression. As in WT mice, attenuated *Igf2* expression on the *maternal allele* (lower row) is associated with histone hyperacetylation (H3K9Ac), DNA hypomethylation (5mC), and increased transcription factor (CTCF) binding to the *H19* ICR; however, enhanced transcription factor (*Aβ*_*42*_) binding to *Igf2* DMR2 is associated with histone hypoacetylation (H3K9Ac), histone hypermethylation (H3K9me3), DNA hypermethylation (5mC) and further attenuation of *Igf2* expression. *In this context, the regions of DNA that bind Aβ*_*42*_* (i.e.,* A*β*ID*, Aβ interacting domains) have repressive chromatin marks and increased Aβ*_*42*_* binding association*. (**b**) Temporal effects of *Aβ*_*42*_ on *Igf2* regulation. In cultured cells: Increased *Aβ*_*42*_ levels are associated with increased binding of *Aβ*_*42*_ to, and DNA hypermethylation of, the *Igf2* differentially methylated region 2 (DMR2) (3-Days). Persistent *Igf2* DMR2* Aβ*_*42*_ binding and DNA hypermethylation combined with histone modifications (deacetylation and methylation) are associated with a stable reduction of *Igf2* expression and IGF2 levels (6-Days, 9-Days). IGF2*/Igf2*, insulin growth factor 2; DMR2, differentially methylated region 2; ICR, imprinting control region; CTCF, CCCTC-binding factor, *Aβ*_*42*_, amyloid beta 1–42.

Our findings demonstrate that IGF2 levels and *Igf2* expression in WT mice is associated with increased histone acetylation, transcription factor (CTCF) binding and *H19* ICR hypomethylation, whereas the reduced IGF2 levels and *Igf2* expression in the cerebrum, liver, and blood plasma of 5xFAD mice is associated with increased histone methylation, transcription factor (*Aβ*_*42*_) binding and *Igf2* DMR2 hypermethylation (Table S1). The results from 1.5-month-old 5xFAD mouse pups (Table S2), which lack *Aβ*_*42*_ accumulation and do not differ from WT mice in IGF2 levels suggest that *Aβ*_*42*_ accumulation precedes or is associated with the genotype- and tissue-specific alterations in the epigenetic regulation of *Igf2* expression and IGF2 levels observed in symptomatic 5xFAD mice. Results from humans diagnosed with AD (Table S3) suggest a similar causal relationship among *Aβ*_*42*_ levels, epigenomic state, and *Igf2* expression in the frontal cortex, reflecting a conserved mechanism for *Igf2* gene regulation in AD (Fig. 7a). In addition, *Aβ*_*42*_ treatment of human derived HEK239 cells (Table S4) induced DNA methylation, histone deacetylation and histone methylation, and a reduction in *Igf2* expression through stable binding of *Aβ*_*42*_ to the *Igf2* DMR2 promoter region (Fig. 7b). Thus, *Aβ*_*42*_ association with the *Igf2* DMR on the *Igf2* promoter provides a nonconical mechanism for reduced IGF2 levels in 5xFAD mice and AD patients, independent of *H19* ICR DNA methylation. These findings suggest a causal relationship among epigenomic state, *Igf2* expression, and IGF2 levels in a mouse model with age-related changes in metabolism, reduced weight gain, tissue degradation, and cognitive/motor decline and provide a potential mechanism for mouse and human *Igf2* gene regulation in normal and pathological conditions.

In agreement with previous reports^68^, our results show reduced IGF2 levels in the CNS of young 5xFAD mice, which further decline with age and lower IGF2 levels in females than males at both 6- and 12-months of age (Fig. 1b). To determine whether attenuated IGF2 levels were due to tissue-specific epigenetic regulation, we measured *H19* ICR methylation, *Igf2* expression and IGF2 levels and found sex- and age-dependent changes in IGF2 protein, as well as epigenetic regulation of the *Igf2/H19* locus in the cerebrum, liver, and plasma of young and aged 5xFAD mice (Fig. 1b–i). To our knowledge, this is the first study to investigate *H19* ICR promoter methylation, *Igf2* expression and IGF2 levels in neural and peripheral tissues of young and aged 5xFAD mice. Overall, the *Igf2* mRNA and IGF2 protein levels were lower in 1) 5xFAD mice than WT mice, 2) females than males, and 3) older mice than younger mice.

These results suggest that it might be important to determine the nature of brain-liver activities and how such interactions result in sustained changes in gene expression and function over the lifespan and in AD. As in the cerebrum, IGF2 levels were significantly lower in the liver of 5xFAD mice compared to the WT mice (Fig. 1c). The qPCR analysis showed decreased *Igf2* mRNA transcript levels in the liver of 5xFAD mice (Fig. 1f), suggesting that IGF2 is endogenously produced in the liver rather than carried in from other tissues. Reduced IGF2 levels in the liver of 5xFAD mice suggests reduced metabolic activity in the liver, as occurs in the mouse^76^ (and human^77^) brain. IGF2 depletion accelerates the onset of metabolic deficits, reduced cell survival, and cognitive impairments by altering growth hormone-related gene expression. Indeed, memory and synaptic deficits in mouse models of AD can be reversed by treatment with IGF2 analogs^26,30^. Likewise, liver *Igf2* expression and plasma IGF2 levels respond to metabolic status and promote tissue survival and proliferation, along with adipocyte signaling^78,79^. Both liver volume and blood flow decrease with age in mice^80^ (and humans^81^). Together with our findings of *Aβ*_*42*_-related reduction of *Igf2* expression, this may help explain the pathophysiology of peripheral metabolic deficits observed in AD mouse models^18^ and humans diagnosed with AD^82^.

There are several CTCF binding sites on the mouse *H19* promoter (Fig. 1a and S1a). We therefore examined the potential mechanism for *Igf2* regulation by CTCF. The 5xFAD mice had lower levels of *Igf2* mRNA and IGF2 in the cerebrum and liver (Fig. 1e,f). However, we did not find any genotype differences in *H19* ICR methylation or CTCF binding (Fig. S1b–d), suggesting that the reduced *Igf2* expression observed in the cerebrum and liver of 5xFAD mice is independent of canonical *H19* imprinting.

A switch from monoallelic to biallelic human *Igf2* promoter methylation during aging has been documented^83^, and this may be partly responsible for the reduced levels seen in rodents and humans^34,84,85^. This is further supported by our results; while there were no age differences in *H19* ICR methylation or CTCF in the cerebrum, IGF2 promoter methylation was higher in the 12-month-old than the 6-month-old mice (Fig. S1b). On the other hand, there was a direct relationship between *H19* and *Igf2* regulation for the sex differences observed in this study: the female mice had lower IGF2 levels than males, accompanied by lower *H19* ICR methylation and higher CTCF binding. In support of this, lower expression levels of IGF2 and *H19*, along with other imprinted genes such as *Peg3* and *Zim1* have previously been reported in female mice, measurable from later stages of pregnancy onwards^86^.

To determine whether attenuated IGF2 levels were associated with AD neuropathology, we measured *Aβ*_*42*_ levels in the cerebrum, liver, and blood plasma of young and aged 5xFAD mice (Fig. 2a–c). To our knowledge, this is the first study to show the presence of *Aβ*, as well as decreased IFG2, in the liver of 5xFAD mice (Fig. 2b). Although the exact source needs to be determined, the *Aβ*_*42*_ found in the hepatic tissue may have come from the circulating *Aβ* that we detected in the plasma (Fig. 2c). Our results show that: 1) *Aβ*_*42*_ levels were higher in the cerebrum and liver of 5xFAD mice than WT mice; 2) the accumulation of *Aβ*_*42*_ begins before 6-months of age; 3) *Aβ*_*42*_ levels increase as the mice continue to age; and that 4) IGF2 levels decrease as *Aβ*_*42*_ levels increase (indicated by reduced IGF2 levels in older 5xFAD mice). These results enhance the validity of the 5xFAD mice as a model for AD, as enriched *Aβ* in peripheral tissues in humans carrying the Swedish mutation has been reported^87^, and shown to slow down *Aβ* clearance in the brain^88^. Indeed, increased peripheral tissue damage (including the liver) in mild AD and cognitive impairment has been linked to ApoE*-ε*4 allele carriers^89^.

We found an *Aβ*_*42*_ binding (*AβID*) region on the mouse *Igf2* promoter (Fig. 3a), implying that the *Igf2* gene may be co-regulated by *Aβ*_*42*_, which has been shown to act as a transcription factor for the *APP*, *β-secretase*, and *ApoE* genes, as well as *Txnip*^5,9^. In support of this theory, *Aβ*_*42*_ selectively associated with genomic DNA sequences that contained the potential *AβID* region, in cerebrum of 12-month-old mice and human frontal cortex as well as ex vivo in human-derived HEK293 cells (compare Figs. 3b, 5b and 6b). We therefore examined the potential mechanism for *Igf2* gene regulation by CTCF and *Aβ*_*42*_. ChIP- and double-ChIP-qPCR assays measuring chromatin modifications showed lower histone acetylation and higher DNA methylation on the *AβID* response element region of the 5xFAD mice, suggesting that cerebrum and liver *Igf2* expression is silenced both by *Aβ*_*42*_ and CTCF (Fig. 3c–k). Our results show that *Aβ*_*42*_ can interact with gene regulatory elements and recruit heterochromatin marks associated with gene silencing (i.e., decreased H3K9Ac and increased H3K9me3) through specific binding to the *AβID* sequence. While consistent with other reports^90^, this further demonstrates that the effect of *Aβ*_*42*_ on transcription (either increasing or decreasing mRNA expression) is gene-specific^5,75^. These results were supported by double ChIP-qPCR assays to study DNA methylation with *Aβ*_*42*_ and CTCF binding (Fig. 3c–e and S1b–d). WT mice had hypomethylated *AβID* and hypermethylated CTCF regions accompanied by low *Aβ*_*42*_ and CTCF binding. Conversely, 5xFAD mice had hypermethylated *AβID* and hypomethylated CTCF regions accompanied by higher *Aβ*_*42*_ and CTCF binding, which explains the low amounts of IGF2 in the cerebrum, liver, and plasma of the 5xFAD mice.

IGF2 reduction contributes to AD pathogenesis due to its role in *Aβ*_*42*_ clearance^91^. Reduced IGF2 levels in the liver could accelerate the disease process. In hepatic cells, during normal aging, IGF2 directly interacts with growth hormone to increase its transcriptional activity for promotion of somatic growth and the regulation of metabolism^92^. The reduction of IGF2 levels in the cerebrum and liver may help to explain the pathological weight loss and cognitive deficits seen in the 5xFAD mice^18,60^. Higher IGF2 levels in the young WT mice may be a part of normal development, protecting developing blood cells against oxidative damage. Since the levels of IGF2 are much lower in the 5xFAD mice than WT mice, this may contribute to blood-related abnormalities, such as higher oxygen consumption^93^ or elevated oxidative damage of DNA by reactive oxygen species, as shown by our findings in 3xTg-AD mice^9^. We found higher levels of *Aβ*_*42*_ binding to a potential *AβID* region of *Igf2* DMR2 (Fig. 3e) along with reduced IGF2 levels (Fig. 1d) in plasma samples of the 12-month-old 5xFAD mice compared to the 6-month-old mice, even though the levels of *Aβ*_*42*_ were lower in the older mice (Fig. 2c). Reductions in plasma *Aβ*_*42*_ levels occur in later stages of AD^94^, and may result in reduced clearance of *Aβ*_*42*_ from the CNS due to formation of large *Aβ* aggregates and plaques or clearance of other toxic species that contribute to AD progression. Higher binding to the *Igf2* promoter, even at lower concentrations, suggest that various *Aβ* species are present in the 5xFAD mice in an age dependent manner and more toxic species may be forming as the mice age. While our ELISA method was not capable of differentiating between these species, which is a limitation of this study, the role of different sizes and shapes of *Aβ* oligomers on different AD mechanisms has been reported^95^.

The canonically imprinted *Igf2*/*H19* locus is conserved between mice and humans in both DNA sequence and epigenetic regulation^52^. To determine whether the findings from the 5xFAD mouse model supported relevance of mechanism to human disease, we compared *Igf2* epigenetic regulation in frontal cortex from aged humans diagnosed with AD and non-AD patients. Our findings show that the levels of IGF2 and *Igf2* mRNA expression were lower in the frontal cortex of AD than non-AD patients and lower in females than males (Fig. 4a,b), with the sex differences driven by *H19* ICR DNA methylation (Fig. 4c with S2b), whereas genotype differences were driven by *Aβ*_*42*_ bound to the *Igf2* DMR2 (Fig. 5c), independent of the levels of *H19* ICR DNA methylation (compare Figs. 5c with 4c and S2b). These results, together with the *Aβ*_*42*_ association with the *Igf2 AβID* region (Fig. 5c), provide evidence for conserved mechanisms for *Igf2* regulation in 5xFAD mice and AD patients. To the best of our knowledge, this the first time *Aβ*_*42*_ has been shown to have a role as a transcription factor and epigenetic regulator in the human brain, altering transcription of an imprinted gene independently of canonical imprinting.

Exogenous *Aβ*_*40*_ and *Aβ*_*42*_ oligomers have been shown to translocate to the cell nucleus in culture^8^, where *Aβ*_*42*_ can bind to genomic DNA and alter gene expression^5,9^. To further characterise the temporal order of events that lead to a decrease in IGF2 and to examine whether *Aβ*_*42*_ bound to the *Igf2* promoter region can directly mediate *Igf2* transcription, HEK293 cultures were treated with *Aβ*_*42*_ oligomers and then harvested at various time points to evaluate temporal changes in *Igf2* epigenetic status and expression.

Our results show exogenous *Aβ*_*42*_ was detectable in the cell nucleus 3-days following *Aβ*_*42*_ treatment, but levels decreased 6- and 9-days following *Aβ*_*42*_ treatment, suggesting leakage or an active clearance mechanism (Fig. 6a). While the endocytosis and nuclear transport mechanisms of *Aβ*_*42*_ have not been fully characterised, these processes have been shown to involve the alpha-7 nicotinic^96^ and toll-like receptors^97,98^. Interestingly, while the nuclear *Aβ*_*42*_ levels decreased over time (Fig. 6a), the levels of *Aβ*_*42*_ bound to DNA did not change (Fig. 6c), suggesting that only the *Aβ*_*42*_ unbound to DNA is cleared from the cell nucleus (compare Fig. 6a,c). In addition, *Igf2* expression and IGF2 levels remained low following *Aβ*_*42*_ treatment (Fig. 6g,h). These results suggest stable association of *Aβ*_*42*_ with the *Igf2* DMR2 may provide a potential mechanism underlying the temporal stability of altered *Igf2* expression patterns in 5xFAD mice and AD.

These results suggest a causal relationship between *Aβ*_*42*_ accumulation, *Aβ*_*42*_ binding and DNA methylation on the *Igf2* DMR2, combined with histone deacetylation and methylation, leading to a stable reduction of *Igf2* expression and IGF2 levels. Similar to our previous findings in 3xTg-AD mice^9^, these results support of the role of *Aβ*_*42*_ as a transcription factor. However, binding of *Aβ*_*42*_ with the *Igf2* DMR *AβID* region results in enhanced histone methylation and *Igf2 silencing* (rather than enhanced histone acetylation and activation as seen with the TXNIP *AβID* region).

### Limitations

While the sample size used in the mouse studies was justified by power analysis, 3 mice per group is still at the lower end. However, the high agreement between the mouse, human, and cell culture results indicates a relatively conserved biological mechanism in mammalian cells and that the findings are biologically valid. Further studies are required to determine the temporal time point(s) of the functional changes in cerebrum and liver of the 5xFAD mice, and how the transgenes in the 5xFAD mice or their derivatives alter brain-liver metabolism, tissue growth, liver function, and the epigenetic status of the *Igf2* gene promoter. In addition, the exact causal relationship between DNA methylation and altered histone acetylation and transcription factor (*Aβ*_*42*_, CTCF) binding remains to be defined, as well as the process by which *Aβ*_*42*_ enters the cell, translocates to the nucleus, and is then cleared. It also remains unclear whether a similar mechanism occurs at *AβID* elements within other imprinted genes or whether neurons and different types of glial cells show the same response. Genetically modified cell lines and animal models with induced disruption to the *Igf2* DMR *AβID* region would permit us to validate the DNA sequence specificity of *Aβ*_*42*_ binding and monitor the dynamics of epigenetic regulation in parallel with behavioral assessments. Future work should also consider the use of allelic-specific primers to examine the possibility of tissue-specific variation in maternal and paternal allele expression, while longitudinal sampling would allow us to determine if these changes are the result of gradual interactions between *Aβ*_*42*_ (and other Aβ species) with AβID regions in neural and non-neuronal cell types.

### Conclusions

This study systematically examined molecular and functional deficits in the cerebrum, liver, and plasma of young and aged 5xFAD mice and showed the transcriptional relationship between *Aβ*_*42*_ and IGF2 in the cerebrum and liver. This transcriptional relationship between *Aβ*_*42*_ and IGF2 was shown to be conserved in the human frontal cortex of individuals diagnosed with AD and was modeled in *Aβ*_*42*_-treated human derived HEK293 cell cultures. Consistently *Aβ*_*42*_ was shown to override gene imprinting and control gene expression. Alterations in histone structure, both in terms of methylation^99^ and acetylation^100^ have been shown in the brains of AD patients, however these changes have been related to tau pathology^101,102^. Thus, our results contribute to the already known role of *Aβ*_*42*_ as a transcription factor, and for the first time confirm it in the human brain. Overall, these findings provide a potential mechanism for the enduring effect of pathological inflammation on brain function in a common chronic progressive neurodegenerative disorder and suggest IGF2 levels could potentially be a useful predictive or diagnostic biomarker for *Aβ*_*42*_-targeted AD therapies.

## Supplementary Information

Supplementary Information.

## Data Availability

The datasets generated during and/or analysed during the current study are available from the corresponding author on reasonable request.
