# Supplementary material for: Noncanonical regulation of imprinted gene Igf2 by amyloid-beta 1–42 in Alzheimer’s disease
Source: Sci Rep. 2023 Feb 4;13:2043. doi: 10.1038/s41598-023-29248-x (PMC9899226; doi:10.1038/s41598-023-29248-x)
Supplement: Supplementary file 1 — Supplementary Information. [file 41598_2023_29248_MOESM1_ESM.pdf]

Supplemental Material for:

Noncanonical regulation of imprinted gene *Igf2* by amyloid-beta 1-42 in Alzheimer's disease

Emre Fertan, William H. Gendron, Aimée A. Wong, Gabrielle M. Hanson, Richard E. Brown, and Ian C.G. Weaver

The supplementary material consists of the following 12 items.

1. Detailed description of the statistical analyses for the data shown in Figure 1. IGF2 levels, *Igf2* expression, and *H19* ICR methylation in the cerebrum, liver, and plasma from 6- and 12-month-old male and female WT and 5xFAD mice.

2. Figure S1, with a detailed description of the statistical analyses for data shown in Figure S1. Epigenetic marks associated with CTCF binding to *H19* ICR in mice.

3. Detailed description of the statistical analyses for data shown in Figure 2. ELISA levels of *Aβ₄₂* in the cerebrum, liver, and plasma of male and female, 5xFAD and WT mice at 6- and 12-months of age.

4. Detailed description of the statistical analyses for data shown in Figure 3. Epigenetic marks associated with $A\beta_{42}$ binding to *Igf2* DMR2 in mice.

5. Table S1. Summary of the analyses of data from 6- and 12-month-old 5xFAD and WT mice in Figures 1, S1 and 2 (* = $p < 0.05$; ** = $p < 0.01$; *** = $p < 0.001$).

6. Table S2. Summary of statistical results from the analyses of 5-week-old 5xFAD and WT mouse data.

7. Detailed description of the statistical analyses for data shown in Figure 4. IGF2 levels, *Igf2* expression, *H19* ICR methylation, and $A\beta_{40}$ and $A\beta_{42}$ levels in the frontal cortex of male and female AD and non-AD patients.

8. Figure S2. Epigenetic marks associated with CTCF binding to *H19* ICR in human frontal cortex.

9. Detailed description of the statistical analyses for data shown in Figure 5. Epigenetic marks associated with $A\beta_{42}$ binding to *Igf2* DMR2 in human frontal cortex.

10. Table S3. Summary of the analyses of data from AD and non-AD patients shown in Figures 4, S2, and 5 (* = $p < 0.05$; ** = $p < 0.01$; *** = $p < 0.001$).

11. Detailed description of the statistical analyses for data shown in Figure 6. Effects of $A\beta_{42}$ on *Igf2* regulation and IGF2 levels in HEK239 cells.

12. Table S4. Summary of the analyses of data from $A\beta_{42}$- and vehicle-treated cell cultures shown in Figure 6 (* = $p < 0.05$; ** = $p < 0.01$; *** = $p < 0.001$).

Supplement 1. Detailed description of the statistical analyses for the data shown in Figure 1. IGF2 levels, *Igf2* expression, and *H19* ICR methylation in the cerebrum, liver, and plasma from 6- and 12-month-old male and female WT and 5xFAD mice. Based on the results of ELISA assays, the levels of IGF2 in the cerebrum were significantly lower in 5xFAD than WT mice ($F_{1, 16} = 8.08$, p = 0.010, $\eta p^2 = 0.288$; **Figure 1b**), significantly lower in females than males ($F_{1, 16} = 11.98$, p < 0.001, $\eta p^2 = 0.375$), and lower in 12-month than 6-month-old mice ($F_{1, 16} = 15.57$, p < 0.001, $\eta p^2 = 0.438$). While this age effect was significant for 5xFAD mice (CI_{95} = 35.658, 1131.438) it was not significant for WT mice (CI_{95} = -33.163, 1420.517). In the liver, there was a genotype by sex by age interaction for IGF2 levels ($F_{1, 16} = 11.77$, p = 0.003, $\eta p^2 = 0.424$; **Figure 1c**); all mice showed a decrease in IGF2 levels at 12-months of age except WT males which had significantly higher levels of IGF2 than all other 12-month-old mice. Circulating IGF2 levels in the blood plasma were significantly lower in 5xFAD than WT mice ($F_{1, 16} = 5.92$, p = 0.025, $\eta p^2 = 0.228$; **Figure 1d**), significantly lower in females than males ($F_{1, 16} = 19.35$, p < 0.001, $\eta p^2 = 0.492$), and significantly lower in 12-month than 6-month-old mice ($F_{1, 16} = 18.26$, p < 0.001, $\eta p^2 = 0.477$). Together, these results show that genotype-, sex- and age-related differences in IGF2 levels occur in the liver and blood plasma as well as in the cerebrum of 5xFAD and WT mice, indicating that IGF2 levels in peripheral tissues and the circulatory system are concomitant with those in the brain.

To determine whether these differences in IGF2 levels were associated with differences in *Igf2* expression, we performed RT-qPCR analyses in cerebrum and liver tissue. *Igf2* mRNA transcript levels in the cerebrum were significantly lower in 5xFAD than WT mice ($F_{1, 16} = 28.97$, p < 0.001, $\eta p^2 = 0.592$; **Figure 1e**), significantly lower in females than males ($F_{1, 16} = 45.23$, p < 0.001, $\eta p^2 = 0.693$) and significantly lower in 12-month than 6-month-old mice ($F_{1, 16} = 37.79$, p < 0.001, $\eta p^2 = 0.654$). The *Igf2* mRNA levels in the liver were significantly lower in 5xFAD than WT mice ($F_{1, 16} = 24.28$, p < 0.001, $\eta p^2 = 0.548$) and lower in females than males ($F_{1, 16} = 69.63$, p < 0.001, $\eta p^2 = 0.77$). There was also a significant genotype by sex by age interaction ($F_{1, 16} = 9.43$, p = 0.007, $\eta p^2 = 0.371$; **Figure 1f**) as the *Igf2* mRNA levels were lower for all mice at 12-months than at 6-months of age, except for the WT males.

In the *H19*/*Igf2* locus, an ICR associated with a DMR2 sequence upstream of *H19* regulates the reciprocal expression of *Igf2* and *H19* (see **Figure 1a**). Hypomethylation of the DMR2 on the maternal allele blocks enhancer driven *Igf2* transcription. To determine whether the differences in *Igf2* expression were associated with alterations in DNA methylation or DNA

hydroxymethylation, we analysed 5mC and 5hmC levels in six distinct loci that are known to be differentially methylated in this region [48-51]. In both 6- and 12-month-old mice, the levels of *H19* ICR methylation (5mC) in the cerebrum were significantly lower in females than males ($F_{1, 16}$ = 57.94, $p < 0.001$, $\eta p^2 = 0.743$; **Figure 1g**), which reflects the reduced *Igf2* expression (**Figure 1e**) and lower IGF2 levels (**Figure 1b**). There was no significant effect of genotype ($F_{1, 16}$ = 3.03, $p = 0.097$) or age ($F_{1, 16}$ = 2.01, $p = 0.171$) on *H19* ICR 5mC levels in the cerebrum, suggesting that mechanisms independent of *H19* ICR methylation status regulate *Igf2* expression in aging 5xFAD mice. The 5hmC levels did not differ between groups (all $p > 0.05$; **Table S1**). The *H19* ICR 5mC levels in the liver were significantly lower in 5xFAD than WT mice ($F_{1, 16}$ = 6.64, p = 0.018, $\eta p^2 = 0.249$; **Figure 1h**), which reflects the reduced *Igf2* expression levels (**Figure 1f**) and lower IGF2 levels (**Figure 1c**). Likewise, levels of *H19* ICR 5mC were significantly lower in the liver from 12-month than 6-month-old mice ($F_{1, 16}$ = 6.30, $p = 0.021$, $\eta p^2 = 0.239$), and significantly lower in the liver of females than males ($F_{1, 16}$ = 44.00, $p < 0.001$, $\eta p^2 = 0.688$). There were no group differences in 5hmC levels (all $p > 0.05$; **Table S1**). The levels of DNA methylation (5mC) on the *H19* ICR of cfDNA in the blood plasma were significantly lower in 5xFAD than in WT mice ($F_{1, 16}$ = 10.28, $p = 0.004$, $\eta p^2 = 0.339$), lower in females than males ($F_{1, 16}$ = 78.94, $p < 0.001$, $\eta p^2 = 0.798$), and lower in 12-month than 6-month-old mice ($F_{1, 16}$ = 10.26, $p < 0.004$, $\eta p^2 = 0.337$; **Figure 1i**). There were no group differences in 5hmC levels (all $p > 0.05$; **Table S1**).

Supplement 2. Figure S1, with a detailed description of the statistical analyses for data shown in Figure S1. Epigenetic marks associated with CTCF binding to *H19* ICR in mice.

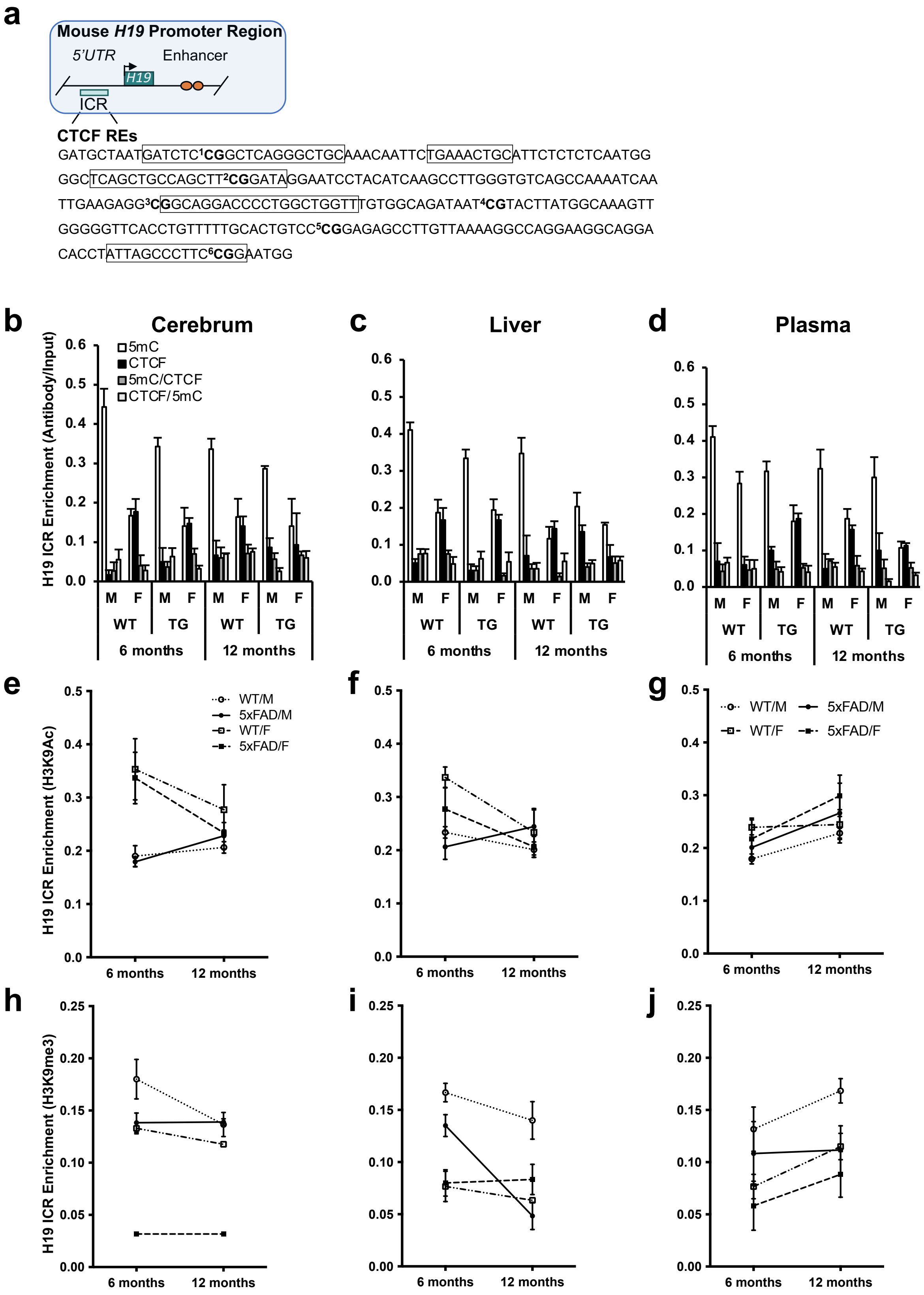

Figure S1. Epigenetic marks associated with CTCF binding to *H19* ICR in mice. Data are expressed as means +/- SEM. (**a**) Schematic representation of the mouse *H19* promotor region (see also **Figure 1a**). Beneath is shown the *H19* imprinting control region (ICR) on the DNA interrogated by chromatin immunoprecipitation (ChIP)-quantitative PCR, with the location of six CpG sites (bold) relative to the CTCF response elements (REs; boxed areas). ChIP-qPCR analysis was performed using antibodies toward 5mC and CTCF with the CTCF binding site on the *H19* ICR in cerebrum, liver, and blood plasma from 6- and 12-month-old male and female 5xFAD and WT mice.

(**b**) The ChIP-qPCR analysis shows that levels of DNA methylation (5mC) on the CTCF binding site on the *H19* ICR in the cerebrum were significantly lower in females than males ($F_{1, 16} = 54.28$, $p < 0.001$, $\eta p^2 = 0.731$), with no effect of genotype ($F_{1, 16} = 3.39$, $p = 0.080$), age ($F_{1, 16} = 2.36$, $p = 0.141$), or genotype by sex interaction ($F_{1, 16} = 0.86$, $p = 0.366$). There was no genotype difference in levels of CTCF association with the CTCF binding site on the *H19* ICR in the cerebrum ($F_{1, 16} = 0.09$, $p = 0.774$) but levels were significantly higher in females than males ($F_{1, 16} = 13.21$, $p = 0.002$, $\eta p^2 = 0.398$), with no effect of age ($F_{1, 16} < 0.01$, $p = 0.943$).

(**c**) Levels of DNA methylation (5mC) on the CTCF binding site on the *H19* ICR in the liver were significantly lower in females than males ($F_{1, 16} = 90.27$, $p < 0.001$, $\eta p^2 = 0.688$), lower in 12-month than 6-month-old mice ($F_{1, 16} = 8.95$, $p = 0.007$, $\eta p^2 = 0.309$) and, while there was no effect of genotype ($F_{1, 16} = 3.09$, $p = 0.097$), there was a genotype by sex interaction ($F_{1, 16} = 9.22$, $p = 0.008$, $\eta p^2 = 0.301$) as 5xFAD males had lower levels than WT males (CI_{Males} = 0.013, 0.207), but females did not differ ($CI_{Females}$ = -0.091, 0.047). Levels of CTCF association with the CTCF binding site on the *H19* ICR in the liver did not differ between genotypes ($F_{1, 16} = 0.08$, $p = 0.780$), but levels were significantly higher in females than males at 6-months of age ($F_{1, 16} = 7.91$, $p = 0.012$, $\eta p^2 = 0.318$), which is predictive of silenced *Igf2* expression and lower IGF2 levels in the younger female mice.

(**d**) Levels of DNA methylation on the CTCF binding site on the *H19* ICR in the plasma were significantly lower in females than males ($F_{1, 16} = 12.61$, $p = 0.002$, $\eta p^2 = 0.387$), and lower in 12-month than 6-month-old females ($F_{1, 16} = 5.43$, $p = 0.030$, $\eta p^2 = 0.214$), with no effect of genotype ($F_{1, 16} = 2.49$, $p < 0.139$). There were no genotype, sex, or age differences in CTCF binding to the *H19* promoter in cfDNA fragments from the plasma (all $p > 0.05$).

The double ChIP assay showed low levels of CTCF association to the methylated CTCF binding site and no significant genotype, sex or age differences in the sequential 5mC/CTCF and

CTCF/5mC immunoprecipitation with the CTCF binding site in cerebrum, liver, and blood plasma (Figure S1b, c, d; all p values > 0.05).

(**e**) The ChIP-qPCR analyses showed no genotype differences in the cerebrum for H3K9Ac association ($F_{1, 16} = 0.22$, $p = 0.641$), however there was a sex by age interaction ($F_{1, 16} = 6.82$, $p = 0.018$, $\eta p^2 = 0.286$); females had higher levels than males ($F_{1, 16} = 14.71$, $p = 0.001$, $\eta p^2 = 0.424$) at 6-months of age ($CI_{6\text{-months}} = 0.082, 0.239$) but not at 12-months ($CI_{12\text{-months}} = -0.098, 0.022$).

(**f**) There was no effect of genotype (F= $F_{1, 16} = 0.64$, $p = 0.433$) for H3K9Ac association with the CTCF binding site on the *H19* ICR in the liver, but there was a significant sex by age interaction ($F_{1, 16} = 5.36$, $p = 0.033$, $\eta p^2 = 0.240$): females had higher levels than males at 6-months of age ($CI_{Sex} = 0.025, 0.149$), but not at 12-months ($CI_{Sex} = -0.062, 0.067$).

(**g**) There were no genotype or sex differences in levels of H3K9Ac association with the CTCF binding site on the *H19* ICR of cfDNA fragments from the blood plasma (all p > 0.05), however, the levels were higher in 12-month than 6-month-old mice ($F_{1, 16} = 5.81$, $p = 0.026$, $\eta p^2 = 0.225$).

(**h**) Levels of H3K9me3 association with the CTCF binding site on the *H19* ICR in the cerebrum revealed a genotype by sex interaction ($F_{1, 16} = 34.38$, $p < 0.001$, $\eta p^2 = 0.669$) and a genotype by age interaction ($F_{1, 16} = 5.53$, $p = 0.031$, $\eta p^2 = 0.245$) as levels for WT female mice were lower than all other groups.

(**i**) Levels of H3K9me3 association with the CTCF binding site on the *H19* ICR in the liver revealed sex by genotype ($F_{1, 16} = 14.01$, $p = 0.002$, $\eta p^2 = 0.452$) and sex by age ($F_{1, 16} = 6.96$, $p = 0.017$, $\eta p^2 = 0.290$) interactions as the male 5xFAD mice showed a decrease in levels between 6- and 12-months of age which was not shown in any other group. Males had higher levels than females at 6-months of age, ($CI_{6\text{-months}} = 0.044, 0.101$) but not at 12 months ($CI_{12\text{-months}} = -0.033, 0.075$).

(**j**) Levels of H3K9me3 association with the CTCF binding site on the *H19* ICR in the blood plasma were lower in 5xFAD than WT mice ($F_{1, 16} = 5.34$, $p = 0.032$, $\eta p^2 = 0.211$) and lower in females than males ($F_{1, 16} = 11.27$, $p = 0.003$, $\eta p^2 = 0.360$). While 12-month-old mice had higher levels than 6-month-old mice, this difference did not reach statistical significance ($F_{1, 16} = 4.01$, $p = 0.059$). Data are expressed as means -/+ SEM. UTR, untranslated region; ICR, imprinting control region; 5mC, 5-methylcytosine; CTCF, CCCTC-binding factor; H3K9Ac, histone 3 lysine-9 acetylation; H3K9me3, histone 3 lysine-9 tri-methylation.

Supplement 3. Detailed description of the statistical analyses for data shown in Figure 2. ELISA levels of *Aβ42* in the cerebrum, liver, and plasma of male and female, 5xFAD and WT mice at 6- and 12-months of age. The results of ELISA analyses show that the levels of *Aβ42* were higher in the cerebrum of 5xFAD than WT mice ($F_{1,16} = 39.7$, $p < 0.001$, $\eta p^2 = 0.661$), and increased with age in 5xFAD but not in WT mice (CI_{5xFAD} = 95.207, 108.642; CI_{WT} = -0.390, 0.253), resulting in an age by genotype interaction ($F_{1,16} = 1135.71$, $p < 0.001$, $\eta p^2 = 0.985$), with no sex difference ($F_{1,16} = 0.03$, $p = 0.868$; **Figure 2a**). Levels of *Aβ42* were higher in the liver of 5xFAD than WT mice ($F_{1,16} = 40.84$, $p < 0.001$, $\eta p^2 = 0.671$), and increased significantly with age in 5xFAD mice (CI_{5xFAD} = 6.073, 37.079) but not in WT mice (CI_{WT} = -2.902, 8.946) resulting in an age by genotype interaction ($F_{1,16} = 15.94$, $p < 0.001$, $\eta p^2 = 0.484$). There was also a sex by age interaction, as levels of *Aβ42* in the liver increased more in males from 6- and 12-months of age than females ($F_{1,16} = 6.92$, $p = 0.018$, $\eta p^2 = 0.289$; **Figure 2b**). The levels of *Aβ42* were higher in the blood plasma of 5xFAD than WT mice ($F_{1,16} = 73.81$, $p < 0 .001$, $\eta p^2 = 0.787$), and decreased with age in 5xFAD mice ($F_{1,16} = 123.86$, $p < 0.001$, $\eta p^2 = 0.879$; CI_{5xFAD} = 24.487, 38.035), but not in WT mice (CI_{WT} = -0.260, 0.410). There was no sex difference in levels of *Aβ42* in the blood plasma ($F_{1,16} = 0.45$, $p = 0.511$; **Figure 2c**).

Supplement 4. Detailed description of the statistical analyses for data shown in Figure 3. Epigenetic marks associated with *Aβ42* binding to *Igf2* DMR2 in mice. Using ChIP-qPCR analyses with an antibody toward *Aβ42* we found that there was significantly greater *Aβ42* association with the *Igf2* DMR2 sequence containing the potential *AβID* region in the cerebrum of 12-month-old 5xFAD mice than there was with the *H19* ICR that did not include this *AβID* region ($t_{3.00} = 5.04$, $p = 0.015$, $d = 3.565$; **Figure 3b**). Levels of DNA methylation on the *AβID* region of *Igf2* DMR2 in the cerebrum showed no genotype difference ($F_{1, 16} = 0.14$, $p = 0.715$), but were significantly higher in the 12-month than 6-month-old mice ($F_{1, 16} = 7.27$, $p = 0.014$, $\eta p^2 = 0.267$), and there was a sex by genotype interaction ($F_{1, 16} = 6.96$, $p = 0.017$, $\eta p^2 = 0.291$). While female WT mice had higher levels of *Igf2* DMR2 methylation than male WT mice (CI_{Sex} = 0.068, 0.289), there was no sex difference in 5xFAD mice (CI_{Sex} = -0.128, 0.065; **Figure 3c**). However, levels of *Aβ42* association with the *AβID* region of *Igf2* DMR2 in the cerebrum were significantly higher in 5xFAD than WT mice ($F_{1, 16} = 42.89$, $p < 0.001$, $\eta p^2 = 0.682$), with no effect of sex or age (all $p > 0.05$; **Figure 3c**). Levels of DNA methylation on the *AβID* region of *Igf2* DMR2 in the liver were significantly higher in 5xFAD than WT mice ($F_{1, 16} = 6.40$, p =

0.020, $\eta p^2 = 0.242$), and were significantly higher in females than males ($F_{1, 16} = 24.70$, $p < 0.001$, $\eta p^2 = 0.553$), with no effect of age ($F_{1, 16} = 0.26$, $p = 0.618$; **Figure 3d**). Levels of *Aβ₄₂* association with the *AβID* region of *Igf2* DMR2 in the liver were significantly higher in 5xFAD than WT mice ($F_{1, 16} = 68.59$, $p < 0.001$, $\eta p^2 = 0.774$), with no effect of sex or age (all $p > 0.05$; **Figure 3d**). There was no effect of genotype on levels of *Igf2* DMR2 methylation of cfDNA fragments from the plasma ($F_{1,16} = 0.30$, $p = 0.593$), but there was a sex by age interaction ($F_{1, 16} = 27.77$, $p < 0.001$, $\eta p^2 = 0.620$): females had lower levels at 12-months than 6-months of age ($CI_{Females} = 0.041, 0.206$), while males had higher levels at 12-months than 6-months of age ($CI_{Males} = 0.064, 0.263$; **Figure 3e**). The 5xFAD mice showed significantly higher *$A\beta_{42}$* binding than WT mice ($F_{1, 16} = 63.30$, $p < 0.001$, $\eta p^2 = 0.760$), and there were genotype by age ($F_{1, 16} = 33.19$, $p < 0.001$, $\eta p^2 = 0.661$) and genotype by sex ($F_{1, 16} = 7.03$, $p = 0.017$, $\eta p^2 = 0.293$) interactions: *$A\beta_{42}$* association with the *Igf2* promoter increased in 5xFAD mice as they aged ($CI_{5xFAD} = 0.041, 0.152$; $CI_{WT} = -0.022, 0.005$), and the increase was greater in 5xFAD females than males (**Figure 3e**), suggesting that cfDNA epigenetic marks at transcription factor binding sites in plasma also reflect age-related AD pathologies. The double ChIP analyses showed low levels *Igf2* DMR2 enrichment in lanes labelled 5mC/*$A\beta_{42}$* and *$A\beta_{42}$*/5mC, with no significant genotype, sex, or age differences in cerebrum, liver, or blood plasma (all p values > 0.05; **Figure 3c-e**).

Levels of H3K9Ac association with the *AβID* region of *Igf2* DMR2 in the cerebrum were significantly lower in 5xFAD than WT mice ($F_{1, 16} = 24.20$, $p < 0.001$, $\eta p^2 = 0.548$), lower in females than males ($F_{1, 16} = 85.97$, $p < 0.001$, $\eta p^2 = 0.811$), and lower in 12-month than 6-month-old mice ($F_{1, 16} = 54.28$, $p < 0.001$, $\eta p^2 = 0.731$; **Figure 3f**). While there was no genotype effect ($F_{1, 16} = 1.09$, $p = 0.309$), there was a significant genotype by sex interaction ($F_{1, 16} = 7.69$, $p = 0.013$, $\eta p^2 = 0.311$) for H3K9Ac association with the *AβID* region of *Igf2* DMR2 in the liver: while males had overall higher levels than females ($F_{1, 16} = 27.29$, $p < 0.001$, $\eta p^2 = 0.557$), the sex difference was only significant within the WT mice ($CI_{WT} = 0.053, 0.219$) and not the 5xFAD mice ($CI_{5xFAD} = -0.175, 0.014$). Moreover, the levels of H3K9Ac association were lower in 12-month than 6-month-old mice ($F_{1, 16} = 29.66$, $p < 0.001$, $\eta p^2 = 0.597$; **Figure 3g**). Levels of H3K9Ac association with the *AβID* region of *Igf2* DMR2 in the blood plasma were also significantly lower in 5xFAD than WT mice ($F_{1, 16} = 6.21$, $p = 0.022$, $\eta p^2 = 0.237$), lower in females than males ($F_{1, 16} = 42.07$, $p < 0.001$, $\eta p^2 = 0.678$), and lower in 12-month than 6-month-old mice ($F_{1, 16} = 33.25$, $p < 0.001$, $\eta p^2 = 0.624$; **Figure 3h**).

The overall levels of H3K9me3 association with the *AβID* region of *Igf2* DMR2 in the cerebrum were higher in 5xFAD than WT mice ($F_{1, 16} = 6.05$, $p = 0.023$, $\eta p^2 = 0.232$), higher in 12-month than 6-month-old mice ($F_{1, 16} = 10.30$, $p = 0.004$, $\eta p^2 = 0.340$), and higher in females than males ($F_{1, 16} = 6.51$, $p = 0.019$, $\eta p^2 = 0.246$), but there was a genotype by sex by age interaction ($F_{1, 16} = 10.95$, $p = 0.004$, $\eta p^2 = 0.406$) as 5xFAD males had a decrease with age whereas all other groups showed an increase with age (**Figure 3i**). In the liver, there was no significant genotype difference for H3K9me3 association ($F_{1, 16} = 0.14$, $p = 0.713$), but there was a sex by age interaction ($F_{1, 16} = 8.67$, $p = 0.009$, $\eta p^2 = 0.338$); females showed an increase from 6- to 12-months of age ($F_{1, 16} = 8.16$, $p = 0.010$, $\eta p^2 = 0.290$; $CI_{Females} = 0.050, 0.211$) but males did not ($CI_{Males} = -0.050, 0.033$; **Figure 3j**). Levels of H3K9me3 association with the *AβID* region of *Igf2* DMR2 in the blood plasma were significantly higher in females than males ($F_{1, 16} = 10.66$, $p = 0.004$, $\eta p^2 = 0.348$), and higher in 12-month than 6-month-old mice ($F_{1, 16} = 18.07$, $p < 0.001$, $\eta p^2 = 0.475$), with no effect of genotype ($F_{1, 16} = 1.99$, $p = 0.174$; **Figure 3k**).

Supplement 5. Table S1. Summary of the analyses of data from 6- and 12-month-old 5xFAD and WT mice in Figures 1, S1, 2, and 3 (* = p < 0.05; ** = p < 0.01; *** = p < 0.001).

Assay	Target	Cerebrum	Liver	Plasma
**ELISA**	**IGF2**	5xFAD < WT *<br>Female < Male ***<br>5xFAD 12m < 5xFAD 6m ***<br>**Figure 1b**	Female < Male *<br>12m < 6m **<br>5xFAD Male 12m < WT Male 12m *<br>**Figure 1c**	5xFAD < WT *<br>Female < Male ***<br>12m < 6m ***<br>**Figure 1d**
**RT-qPCR**	***Igf2*** **mRNA**	5xFAD < WT ***<br>Female < Male ***<br>12m < 6m ***<br>**Figure 1e**	5xFAD < WT ***<br>Female < Male ***<br>12m < 6m *<br>WT Male 12m = WT Male 6m<br>**Figure 1f**	
**GlucMS-qPCR**	***H19*** **ICR 5mC**	5xFAD = WT<br>Female < Male ***<br>12m = 6m<br>**Figure 1g**	5xFAD < WT *<br>Female < Male ***<br>12m < 6m *<br>**Figure 1h**	5xFAD < WT **<br>Female < Male ***<br>12m < 6m **<br>**Figure 1i**
**GlucMS-qPCR**	***H19*** **ICR 5hmC**	5xFAD = WT<br>Female = Male<br>12m = 6m<br>**Not shown**	5xFAD = WT<br>Female = Male<br>12m = 6<br>**Not shown**	5xFAD = WT<br>Female = Male<br>12m = 6m<br>**Not shown**
**Chip-qPCR**	***H19*** **ICR 5mC**	5xFAD = WT<br>Female < Male ***<br>12m = 6m<br>**Figure S1b**	5xFAD Male < WT Male**<br>Female < Male ***<br>12m < 6m **<br>**Figure S1c**	5xFAD = WT<br>Female < Male **<br>12m Female < 6m Female *<br>**Figure S1d**
**Chip-qPCR**	***H19*** **ICR CTCF**	5xFAD = WT<br>Female > Male **<br>12m = 6m<br>**Figure S1b**	5xFAD = WT<br>6m Female > Male 6m *<br>**Figure S1c**	5xFAD = WT<br>Female = Male<br>12m = 6m<br>**Figure S1d**
**Chip-qPCR**	***H19*** **ICR H3K9Ac**	5xFAD = WT<br>Male 6m < Female 6m *<br>**Figure S1e**	5xFAD = WT<br>Female 6m > Male 6m *<br>**Figure S1f**	5xFAD = WT<br>Female = Male<br>12m > 6m *<br>**Figure S1g**
**Chip-qPCR**	***H19*** **ICR H3K9me3**	5xFAD Female < WT Female **<br>5xFAD 6m < WT 6m *<br>**Figure S1h**	WT Female < WT Male **<br>Female 6m < Male 6m *<br>**Figure S1i**	5xFAD < WT *<br>Female < Male **<br>12m = 6m<br>**Figure S1j**
**ELISA**	***Aβ42***	5xFAD > WT ***<br>Female = Male<br>5xFAD 12m > 5xFAD 6m ***<br>**Figure 2a**	5xFAD > WT ***<br>Female < Male *<br>5xFAD 12m < 5xFAD 6m ***<br>**Figure 2b**	5xFAD > WT ***<br>Female = Male<br>5xFAD 12m < 5xFAD 6m ***<br>**Figure 2c**
**Chip-qPCR**	***H19*** **ICR verses** ***Igf2*** **DMR2** ***Aβ42***	*Igf2* DMR2 > H19 ICR *<br>*Igf2* DMR2, 5xFAD > WT *<br>**Figure 3b**		
**Chip-qPCR**	***Igf2*** **DMR2 5mC**	5xFAD = WT<br>WT Female > WT Male *<br>12m > 6m *<br>**Figure 3c**	5xFAD > WT *<br>Female > Male **<br>12m = 6m<br>**Figure 3d**	5xFAD = WT<br>Male 12m > Male 6m *<br>Female 12m < Female 6m *<br>**Figure 3e**
**Chip-qPCR**	***Igf2*** **DMR2** ***Aβ42***	5xFAD > WT ***<br>Female = Male<br>12m = 6m<br>**Figure 3c**	5xFAD > WT ***<br>Female = Male<br>12m = 6m<br>**Figure 3d**	5xFAD > WT ***<br>5xFAD 12m > 5xFAD 6m ***<br>5xFAD Female > 5xFAD Male ***<br>**Figure 3e**
**Chip-qPCR**	***Igf2*** **DMR2 H3K9Ac**	5xFAD < WT ***<br>Female < Male ***<br>12m < 6m ***<br>**Figure 3f**	5xFAD = WT<br>WT Female < WT male ***<br>12m < 6m ***<br>**Figure 3g**	5xFAD < WT *<br>Female < Male ***<br>12m < 6m ***<br>**Figure 3h**
**Chip-qPCR**	***Igf2*** **DMR2 H3K9me3**	5xFAD > WT *<br>Female > Male *<br>5xFAD Male 6m > WT Male 6m **<br>12m > 6m **<br>**Figure 3i**	5xFAD = WT<br>Female 12m > Female 6m **<br>**Figure 3j**	5xFAD = WT<br>Female > Male **<br>12m > 6m ***<br>**Figure 3k**

Supplement 6. Table S2. Summary of statistical results from the analyses of 5-week-old 5xFAD and WT mouse data.

		Cerebrum			Liver			Plasma		
**Assay**	**Target**	**DoF**	**p value**	**95% CI**	**DoF**	**p value**	**95% CI**	**DoF**	**p value**	**95% CI**
**ELISA**	**IGF2**	3.59	0.757	-507.94, 639.74	3.90	0.889	-401.18, 446.18	3.96	0.924	-590.29, 548.99
**RT-qPCR**	***Igf2* mRNA**	3.49	0.750	-1.96, 2.49	3.48	0.831	-2.25, 2.63	3.97	0.916	-2.65, 2.88
**GlucMS-qPCR**	***H19* ICR 5mC**	4.00	0.745	-8.96, 6.96	3.67	0.917	-8.24, 8.91	2.31	1.00	-8.21, 8.21
**GlucMS-qPCR**	***H19* ICR 5hmC**	3.99	0.851	-4.96, 4.30	2.88	0.819	-4.02, 4.68	2.62	0.702	-6.08, 4.75
**Chip-qPCR**	***H19* ICR 5mC**	4.00	0.931	-0.097. 0.104	3.56	0.463	0.043, 0.076	3.95	0.723	-0.111. 0.084
**Chip-qPCR**	***H19* ICR CTCF**	2.86	1.00	-0.07, 0.07	3.86	1.000	-0.09, 0.09	3.30	0.755	-0.10, 0.08
**Chip-qPCR**	***H19* ICR H3K9Ac**	3.39	0.949	-0.09, 0.09	3.10	0.867	-0.08, 0.09	3.98	0.612	-0.09, 0.06
**Chip-qPCR**	***H19* ICR H3K9me3**	3.96	0.927	-0.14, 0.15	3.77	0.804	-0.10, 0.12	3.10	0.410	-0.07, 0.04
**ELISA**	***Aβ40***	2.86	0.404	-0.18, 0.10	3.66	0.506	-0.08, 0.14	3.48	0.432	-0.07, 0.12
**ELISA**	***Aβ42***	3.60	0.405	-0.04, 0.08	3.48	0.552	-0.17, 0.11	2.35	0.965	-0.15, 0.15
**Chip-qPCR**	***Igf2* DMR2 5mC**	2.44	0.477	-0.16, 0.25	3.08	0.621	-0.14, 0.20	3.94	0.957	-0.17, 0.16
**Chip-qPCR**	***Igf2* DMR2 *Aβ42***	4.00	0.101	-0.02, 0.01	3.2	1.000	-0.04, 0.04	2.94	0.686	-0.02, 0.03
**Chip-qPCR**	***Igf2* DMR2 H3K9Ac**	3.12	0.491	-0.12, 0.21	3.54	0.604	-0.14, 0.21	3.88	0.942	-0.20, 0.21
**Chip-qPCR**	***Igf2* DMR2 H3K9me3**	3.64	0.201	-0.10, 0.03	2.58	0.273	-0.16, 0.07	4.00	0.699	-0.10, 0.08

Supplement 7. Detailed description of the statistical analyses for data shown in Figure 4. IGF2 levels, *Igf2* expression, *H19* ICR methylation, and $A\beta_{40}$ and $A\beta_{42}$ levels in the frontal cortex of male and female AD and non-AD patients. IGF2 levels in the frontal cortex were lower in AD than non-AD patients ($F_{1, 8} = 5.84$, $p = 0.039$, $\eta p^2 = 0.393$) and lower in females than males ($F_{1, 8} = 15.05$, $p = 0.004$, $\eta p^2 = 0.626$; **Figure 4a**). In agreement with this, *Igf2* mRNA levels in the frontal cortex were lower in AD than non-AD patients ($F_{1, 8} = 13.52$, $p = 0.005$, ηp^2 = 0. 600), and lower in females than males ($F_{1, 8} = 22.75$, $p = 0.001$, $\eta p^2 = 0.717$; **Figure 4b**).

The levels of *H19* ICR 5mC in the frontal cortex were significantly lower in females than males ($F_{1, 8} = 11.70$, $p = 0.008$, $\eta p^2 = 0.565$; **Figure 4c**), but did not differ between AD and non-AD patients ($F_{1, 8} = 4.52$, $p = 0.062$). Levels of *H19* ICR 5hmC did not differ between the disease conditions or sexes (all $p > 0.05$; **Table S3**). Results of ELISA analyses showed that AD patients had higher levels of $A\beta_{40}$ ($F_{1, 8} = 82.90$, $p < 0.001$, $\eta p^2 = 0.902$; **Figure 4d**), $A\beta_{42}$ ($F_{1, 8} = 55.03$, $p < 0.001$, $\eta p^2 = 0.859$; **Figure 4e**), and a higher $A\beta_{42}/A\beta_{40}$ ratio ($F_{1, 8} = 53.55$, $p < 0.001$, ηp^2 = 0.856; **Figure 4f**) than non-AD patients. There were no sex differences in any of these measures (all $p > 0.05$; **Table S3**).

Supplement 8. Figure S2. Epigenetic marks associated with CTCF binding to *H19* ICR in human frontal cortex.

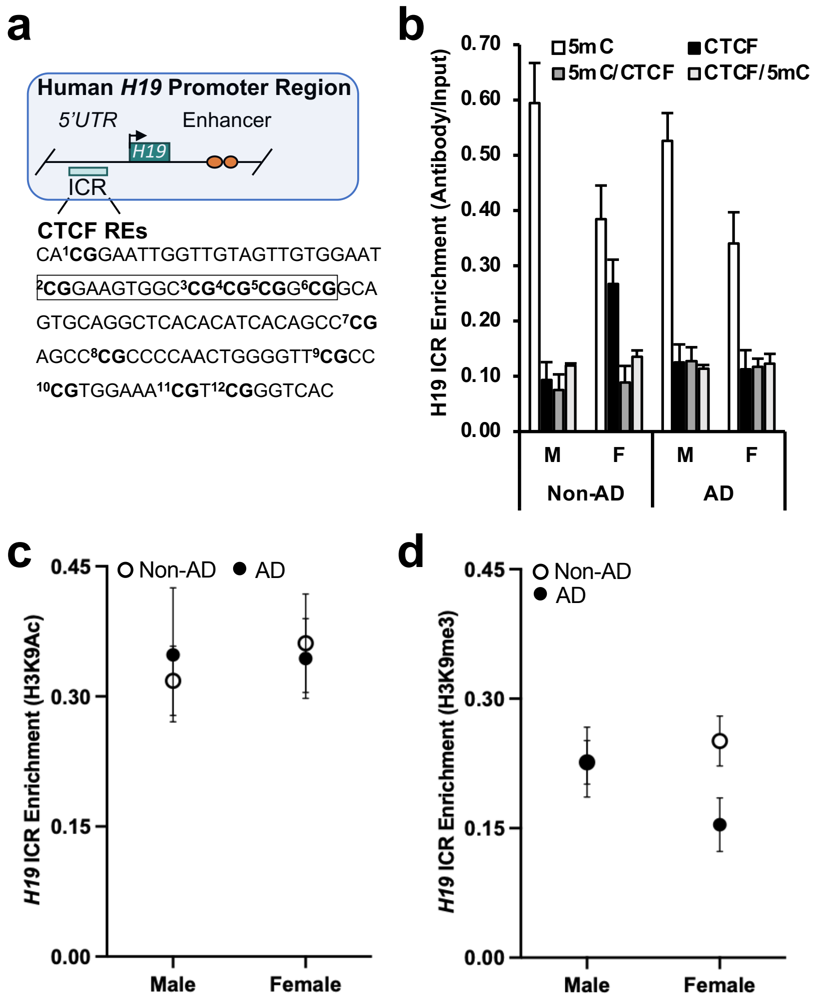

Figure S2. Epigenetic marks associated with CTCF binding to *H19* ICR in human frontal cortex. Data are expressed as means +/- SEM. (**a**) Schematic representation of the human *H19* promotor region (see also **Figure 1a**). Beneath is shown the *H19* ICR on the DNA interrogated by ChIP-qPCR, with the location of 12 CpG sites (bold) relative to the CTCF response elements (REs; boxed areas).

(**b**) The Chip-qPCR analyses showed that levels of DNA methylation (5mC) on the CTCF binding site on the *H19* ICR (5mC)in the frontal cortex were significantly lower in females than males ($F_{1, 16} = 11.93$, $p < 0.007$, $\eta p^2 = 0.570$), with no effect of AD state ($F_{1, 16} = 0.96$, $p < 0.352$). While there was no main effect of disease condition ($F_{1, 8} = 1.80$, $p = 0.213$) or sex ($F_{1, 8} = 3.07$, $p = 0.114$), there was a significant disease by sex interaction ($F_{1, 8} = 6.65$, $p = 0.033$, $\eta p^2 = 0.454$): levels of CTCF association with the CTCF binding site on the *H19* ICR in

the frontal cortex were significantly higher in non-AD females than non-AD males (CI_{Sex} = 0.023, 0.324), but there was no sex difference in CTCF binding in the frontal cortex from AD patients (CI_{Sex} = -0.120, 0.145). The double ChIP assay showed low levels *H19* ICR enrichment in lanes labelled 5mC/CTCF and CTCF/5mC, with no differences between AD and non-AD patients and no sex difference (all p values > 0.05).

There were no significant genotype or sex differences between groups in histone acetylation (**c**) or methylation (**d**; all p > 0.05). Data are expressed as means -/+ SEM. UTR, untranslated region; ICR, imprinting control region; 5mC, 5-methylcytosine; CTCF, CCCTC-binding factor; H3K9Ac, histone 3 lysine-9 acetylation; H3K9me3, histone 3 lysine-9 tri-methylation.

Supplement 9. Detailed description of the statistical analyses for data shown in Figure 5. Epigenetic marks associated with *$A\beta_{42}$* binding to *Igf2* DMR2 in human frontal cortex. Using ChIP-qPCR analyses with an antibody toward *$A\beta_{42}$* we found that there was significantly greater *$A\beta_{42}$* association with the *Igf2* DMR2 sequence containing the potential *AβID* region in frontal cortex of AD patients than there was with the *H19* ICR that did not include this *AβID* region ($t_{3.03}$ = 5.08, p = 0.014, *d* = 3.593; **Figure 5b**). Using ChIP-qPCR analyses with antibodies toward 5mC and *$A\beta_{42}$* we found that levels of *Igf2* DMR2 methylation (5mC) in the frontal cortex were not significantly different between AD and non-AD patients ($F_{1, 8}$ = 0.09, p = 0.771), but were significantly higher in females than males ($F_{1, 8}$ = 8.13, p = 0.019, ηp^2 = 0.475; **Figure 5c**). Conversely, *$A\beta_{42}$* association with *Igf2* DMR2 was significantly higher in the frontal cortex from AD compared to non-AD patients ($F_{1, 8}$ = 13.30, p = 0.005, ηp^2 = 0.596), but there was no sex difference ($F_{1, 8}$ = 1.50, p = 0.253; **Figure 5c**). Similar to the results in mice, the double ChIP assays demonstrated that *$A\beta_{42}$* did not strongly bind to methylated DNA (evidenced by low levels *Igf2* DMR2 enrichment in lanes labelled 5mC/*$A\beta_{42}$* and *$A\beta_{42}$*/5mC; **Figure 5c**). The ChIP-qPCR analyses measuring H3K9Ac and H3K9me3 within the *AβID* region found that the levels H3K9Ac association with *Igf2* DMR2 in the frontal cortex were higher in female than male non-AD patients ($F_{1, 8}$ = 7.91, p = 0.023, ηp^2 = 0.497; $CI_{non\text{-}AD}$ = 0.089, 0.615) but not in AD patients (CI_{AD} = -0.123, 0.201; **Figure 5d**). Conversely, H3K9me3 association with *Igf2* DMR2 in the frontal cortex was significantly higher in AD than non-AD patients ($F_{1, 8}$ = 7.05, p = 0.026, ηp^2 = 0.439), with no sex difference ($F_{1, 8}$ = 3.84, p = 0.082; **Figure 5e**).

Supplement 10. Table S3. Summary of the analyses of data from AD and non-AD patients shown in Figures 4, S2, and 5 (* = p < 0.05; ** = p < 0.01; *** = p < 0.001).

Assay	Target	Frontal Cortex
**ELISA**	**IGF2**	AD < Non-AD *<br>Female < Male **<br>**Figure 4a**
**RT-qPCR**	***Igf2* mRNA**	AD < Non-AD **<br>Female < Male ***<br>**Figure 4b**
**GlucMS-qPCR**	***H19* ICR 5mC**	AD = Non-AD<br>Female < Male **<br>**Figure 4c**
**GlucMS-qPCR**	***H19* ICR 5hmC**	AD = Non-AD<br>Female = Male<br>**Not shown**
**ELISA**	***Aβ40***	AD > Non-AD ***<br>Female = Male<br>**Figure 4d**
**ELISA**	***Aβ42***	AD > Non-AD ***<br>Female = Male<br>**Figure 4e**
**ELISA**	***Aβ42/Aβ40* Ratio**	AD > Non-AD ***<br>Female = Male<br>**Figure 4f**
**Chip-qPCR**	***H19* ICR 5mC**	AD = Non-AD<br>Female < Male **<br>**Figure S2b**
**Chip-qPCR**	***H19* ICR CTCF**	AD = Non-AD<br>AD Female = AD Male<br>Non-AD Female > Non-AD Male *<br>**Figure S2b**
**Chip-qPCR**	***H19* ICR H3K9AC**	AD = Non-AD<br>Female = Male<br>**Figure S2c**
**Chip-qPCR**	***H19* ICR H3K9me3**	AD = Non-AD<br>Female = Male<br>**Figure S2d**
**Chip-qPCR**	***H19* ICR verses *Igf2* DMR2 *Aβ42***	*Igf2* DMR2 > H19 ICR *<br>*Igf2* DMR2, AD > Non-AD *<br>**Figure 5b**
**Chip-qPCR**	***Igf2* DMR2 5mC**	AD = Non-AD<br>Female > Male *<br>**Figure 5c**
**Chip-qPCR**	***Igf2* DMR2 *Aβ42***	AD > Non-AD **<br>Female = Male<br>**Figure 5c**
**Chip-qPCR**	***Igf2* DMR2 H3K9AC**	AD Female = AD Male<br>Non-AD Female > Non-AD Male *<br>**Figure 5d**
**Chip-qPCR**	***Igf2* DMR2 H3K9me3**	AD > Non-AD *<br>Female = Male<br>**Figure 5e**

Supplement 11. Detailed description of the statistical analyses for data shown in Figure 6. Effects of *Aβ42* on *Igf2* regulation and IGF2 levels in HEK239 cells. Results of the ELISA analyses found that levels of *Aβ42* in the cell nuclear fractions were higher in the *Aβ42*-treated than the vehicle-treated HEK293 cells as a function of the number of days the cells spent in culture following *Aβ42* treatment ($F_{2, 18} = 11.95$, $p < 0.001$, $\eta p^2 = 0.570$; **Figure 6a**). Three-days following treatment, the magnitude of the difference in *Aβ42* levels between *Aβ42*- and vehicle-treated cultures was greatest ($CI_{A\beta42\ \mathrm{Treatment}} = 2.888, 9.443$). While still significant, 6-days after treatment the magnitude of the difference in *Aβ42* levels between *Aβ42*- and vehicle-treated cultures was reduced ($CI_{A\beta42\ \mathrm{Treatment}} = 0.368, 3.333$), and was not significant 9-days following *Aβ42* treatment ($CI_{A\beta42\ \mathrm{Treatment}} = -1.129, 0.283$; **Figure 6a**). These results suggest that exogenous *Aβ42* can enter the cell nucleus, followed by temporal removal (clearing). The ChIP-qPCR analyses with an antibody toward *Aβ42* showed that in the *Aβ42* treated cultures, there was significantly greater *Aβ42* binding with *Igf2* DMR2 than there was with *H19* ICR ($t_{3.00} = 8.04$, $p = 0.004$, $d = 5.682$), while there was no difference in the vehicle-treated cultures (**Figure 6b**). The levels of *Aβ42* binding to *Igf2* DMR2 were significantly higher in *Aβ42*-treated cultures than control cultures ($F_{1, 18} = 75.00$, $p < 0.001$, $\eta p^2 = 0.709$) at all time points (**Figure 6c**), as were the levels of *Igf2* DMR2 DNA methylation ($F_{1, 18} = 46.97$, $p < 0.001$, $\eta p^2 = 0.701$; **Figure 6d**).

Levels of *Igf2* DMR2 histone acetylation were decreased in the *Aβ42*-treated cultures compared to control cultures ($F_{1, 18} = 5.73$, $p = 0.027$, $\eta p^2 = 0.223$; **Figure 6e**), while levels of *Igf2* DMR2 histone methylation increased as a function of the number of days the cells spent in culture following *Aβ42* treatment ($F_{2, 18} = 4.73$, $p = 0.022$, $\eta p^2 = 0.253$, **Figure 6f**). The levels of *Igf2* DMR2 histone methylation in *Aβ42*-treated and vehicle-treated cultures were not significantly different after 3-days of *Aβ42* treatment ($CI_{A\beta42\ \mathrm{Treatment}} = -0.105, 0.074$), while after 6- ($CI_{A\beta42\ \mathrm{Treatment}} = 0.036, 0.372$) and 9-days ($CI_{A\beta42\ \mathrm{Treatment}} = 0.110, 0.292$) they were significantly higher in *Aβ42*-treated than control cultures. Levels of *Igf2* mRNA were significantly lower in *Aβ42*-treated cultures at 6- ($CI_{A\beta42\ \mathrm{Treatment}} = 0.250, 1.470$) and 9-days ($CI_{A\beta42\ \mathrm{Treatment}} = 0.065, 1.338$), but not at 3-days ($CI_{A\beta42\ \mathrm{Treatment}} = -0.151, 1.192$) following *Aβ42* treatment (**Figure 6g**).

Likewise, levels of IGF2 were significantly lower in *Aβ42*-treated than control cultures ($F_{1, 18} = 27.97$, $p < 0.001$, $\eta p^2 = 0.535$) at 6- ($CI_{A\beta42\ \mathrm{Treatment}} = 97.116, 537.624$) and 9-days ($CI_{A\beta42\ \mathrm{Treatment}} = 80.767, 633.715$), but not at 3-days ($CI_{A\beta42\ \mathrm{Treatment}} = -89.497, 500.705$) after *Aβ42* treatment (**Figure 6h**).

Supplement 12. Table S4. Summary of the analyses of data from *Aβ₄₂*- and vehicle-treated cell cultures shown in Figure 6 (* = p < 0.05; ** = p < 0.01; *** = p < 0.001).

Assay	Target	HEK293 Cells
**ELISA**	**Nuclear *Aβ42***	Day-3, *Aβ42*-treated >>> Vehicle ***<br>Day-6, *Aβ42*-treated > Vehicle **<br>Day-9, *Aβ42*-treated = Vehicle<br>**Figure 6a**
**Chip-qPCR**	***H19* ICR verses *Igf2* DMR2 *Aβ42***	*Igf2* DMR2 > H19 ICR *<br>*Igf2* DMR2, *Aβ42*-treated < Vehicle *<br>**Figure 6b**
**Chip-qPCR**	***Igf2* DMR2 *Aβ42***	*Aβ42*-treated > Vehicle ***<br>Day-3 = Day-6 = Day-9<br>**Figure 6c**
**Chip-qPCR**	***Igf2* DMR2 5mC**	*Aβ42*-treated > Vehicle **<br>Day-3 = Day-6 = Day-9<br>**Figure 6d**
**Chip-qPCR**	***Igf2* DMR2 H3K9Ac**	*Aβ42*-treated < Vehicle *<br>Day-3 = Day-6 = Day-9<br>**Figure 6e**
**Chip-qPCR**	***Igf2* DMR2 H3K9me3**	Day-3, *Aβ42*-treated = Vehicle<br>Day-6, *Aβ42*-treated > Vehicle *<br>Day-9, *Aβ42*-treated > Vehicle *<br>**Figure 6f**
**RT-qPCR**	***Igf2* mRNA**	Day-3, *Aβ42*-treated = Vehicle<br>Day-6, *Aβ42*-treated > Vehicle **<br>Day-9, *Aβ42*-treated > Vehicle *<br>**Figure 6g**
**ELISA**	**IGF2**	Day-3, *Aβ42*-treated = Vehicle<br>Day-6, *Aβ42*-treated < Vehicle *<br>Day-9, *Aβ42*-treated < Vehicle *<br>**Figure 6h**
